# Interleukin-1α associates with the tumor suppressor p53 following DNA damage

**DOI:** 10.1038/s41598-020-63779-x

**Published:** 2020-04-24

**Authors:** J. Novak, B. Zamostna, V. Vopalensky, M. Buryskova, L. Burysek, D. Doleckova, M. Pospisek

**Affiliations:** 1 Department of Genetics and Microbiology, Faculty of Science, Charles University, Prague, Czech Republic; 2 Protean s.r.o., Dobra Voda u Ceskych Budejovic, Czech Republic

**Keywords:** Interleukins, Double-strand DNA breaks, Tumour-suppressor proteins

## Abstract

Interleukin-1α (IL-1α) is a dual-function proinflammatory mediator. In addition to its role in the canonical IL-1 signaling pathway, which employs membrane-bound receptors, a growing body of evidence shows that IL-1α has some additional intracellular functions. We identified the interaction of IL-1α with the tumor suppressor p53 in the nuclei and cytoplasm of both malignant and noncancerous mammalian cell lines using immunoprecipitation and the *in situ* proximity ligation assay (PLA). This interaction was enhanced by treatment with the antineoplastic drug etoposide, which suggests a role for the IL-1α•p53 interaction in genotoxic stress.

## Introduction

The tumor suppressor p53 lies at the heart of cellular signaling networks, and orchestrates a number of physiological responses to DNA damage, viral infection, oncogenic transformation, cytokine signaling, cell adhesion or environmental stresses. As a consequence of these events, the ubiquitin-mediated degradation of p53 is attenuated, which facilitates p53 tetramer translocation through nuclear pore complexes^1^. The accumulation and stabilization of p53 in the cell nucleus promotes the transcriptional activation function of p53, resulting in the induction of downstream genes^2^. In this way, DNA repair, the generation of ATP via mitochondrial oxidative phosphorylation, permanent cell cycle arrest or programmed cell death can be initiated^3–6^. Conversely, in unstressed cells, p53 is present at low levels in the cytoplasm, where it is degraded rapidly following synthesis due to its interaction with its natural inhibitor, the E3 ubiquitin ligase Mdm2^7,8^. p53 can be induced by a large number of agents, including UV light, oxidative stress or various DNA-damaging chemicals. Treatment with the topoisomerase II inhibitor etoposide, which is widely used as an antineoplastic drug, leads to p53 induction, stabilization and translocation to both the nucleus and mitochondria^9,10^. Mdm2 synthesis is inhibited in etoposide–treated cells, which blocks the autoregulatory p53–Mdm2 feedback loop^11^, leading to p53 phosphorylation^12,13^ and the consequent upregulation of proteins implicated in cell cycle control and apoptosis.

Interleukin-1α (IL-1α), a pleiotropic cytokine governing defense and inflammatory processes in higher eukaryotes, can act extracellularly and trigger signal transduction pathways through the membrane IL-1 receptor type I (IL1RI). However, the presence of a nuclear localization signal within the amino acid sequence of IL-1α^14^ as well as the absence of a classical signal peptide leader sequence suggest that IL-1α plays a role in the cell nucleus. Indeed, both the 31-kDa IL-1α precursor and its 17-kDa N-terminal peptide (NTP), which is generated by calpain cleavage in the process of IL-1α maturation, are frequently found in the nuclei of eukaryotic cells, whereas mature IL-1α remains in the cytoplasm. Interestingly, as the IL-1α N–terminal domains among different species exhibit striking sequence similarity, IL-1α NTP is most likely not only a byproduct of IL-1α maturation^15^. Various biological properties have been attributed to nuclear IL-1α, including its interaction with the growth suppressor protein necdin^16^ and proteins involved in RNA processing^17^, transactivation of transcription via association with histone acetyltransferase complexes^18–20^, binding to chromatin during apoptosis^21^ and induction of proinflammatory mediators independently on the surface receptors^22,23^. In a recent study, nuclear IL-1α was also reported to promote the proliferation of T cell-derived acute lymphoblastic leukemia cells, presumably through the activation of NF-κB^24^. When IL-1α is aberrantly expressed, its proinflammatory activities underlie the pathogenesis of a vast array of diseases, including rheumatoid arthritis, diabetes mellitus, autoimmune encephalomyelitis, systemic sclerosis, malignant skin diseases and cardiovascular diseases^25–32^. Tight control and the well-balanced regulation of IL-1α activity inside the cell as well as in the extracellular space are therefore crucial.

Cohen *et al*. showed that nuclear IL-1α can localize to DNA damage sites and act as an intracellular DNA damage sensor, reporting defects in chromatin integrity to surrounding tissue cells^33^. In the present study, we identified the previously unreported interaction of IL-1α with the tumor suppressor p53 in both malignant and noncancerous mammalian cell lines and provide evidence that IL-1α can closely colocalize with p53 in both the nucleus and cytoplasm of mammalian cells. Furthermore, this interaction was enhanced by treatment with the DNA–damaging drug etoposide.

## Results

### p53 and IL-1α colocalize in both the cell nucleus and cytoplasm

**Figure 1:**
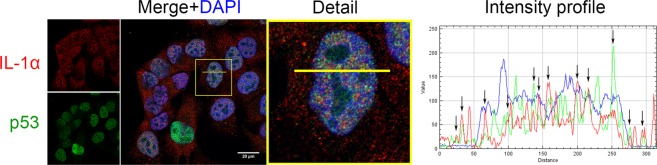
Colocalization of IL-1α and p53 in A375 cells. Endogenous IL-1α (red) and p53 (green) were visualized by indirect immunofluorescence in untreated A375 cells. Nuclei were stained with DAPI. IL-1α localized in both the cytoplasm and cell nucleus, while p53 was present mainly in the cell nucleus. Both proteins were mostly absent from nucleoli. The intensity profile shows the fluorescence intensity of both labeled proteins in the region delineated by the yellow line. Regions showing remarkable colocalization of p53 and IL-1α are marked with arrows. Distance is depicted in pixels. The intensity profile shows overlap of the green and red signals in both the nucleus and cytoplasm. Images were captured with a Zeiss LSM 880 confocal microscope; the scale bar represents 20 µm.

To analyze the subcellular distribution of IL-1α and p53 in mammalian cells and their potential to colocalize, we employed the A375 human malignant melanoma cell line. Endogenous proteins were observed using indirect immunofluorescence and confocal microscopy. Whereas a nuclear distribution of IL-1α and p53 was predominant in our experiments, some signal corresponding to these proteins was regularly detected in the cytoplasm. Data analysis using the ImageJ RGB profiler plugin suggested that a fraction of the p53 and IL-1α populations colocalize in certain regions (Fig. 1). The Pearson’s colocalization coefficient computed using the Coloc2 Fiji plugin also suggested the modest colocalization (r = 0.464) of both proteins. We observed the similar behavior of both proteins in the HeLa human cervical cancer cell line and U2OS human osteosarcoma cell line as well (Supplementary Fig. 1).

### IL-1α coimmunoprecipitates with p53/EGFP from human A375 and Mrc-5 cells

**Figure 2:**
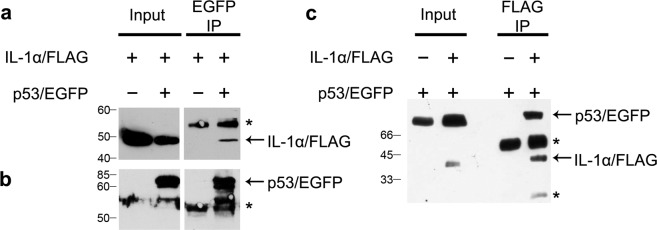
Coimmunoprecipitation of IL-1α and p53. (**a**) A375 cells were transiently cotransfected with plasmids encoding Flag-tagged IL-1α (IL-1α/Flag) and EGFP-tagged p53 (p53/EGFP). Coimmunoprecipitation (IP) was performed using anti-EGFP antibody and transiently produced full-length IL-1α/Flag was detected with anti-Flag antibody; (**b**) the membrane was stripped and p53/EGFP was detected with CM-1 anti-p53 antibody. (**c**) The same experiment was performed in Mrc-5 cells, except that coimmunoprecipitation was performed with anti-Flag antibody. Mouse monoclonal anti-Flag and anti-p53 antibodies were used for the simultaneous detection of full-length IL-1α/Flag and p53/EGFP, respectively. Mock IP was performed in cells not expressing the respective bait. Each panel (a–c) corresponds to a single WB membrane. Asterisks represent antibody heavy and light chains. Numbers correspond to the MW of protein standards. Full size records from these membranes are available in Supplementary Fig. 5.

Endogenous IL-1α and p53 showed similar distribution patterns in confocal microscopy experiments. Therefore, we wanted to test whether they could localize to the same macromolecular complex. To test this possibility, we transiently cotransfected A375 human melanoma cells with recombinant vectors encoding Flag-tagged full-length IL-1α (IL-1α/Flag) and EGFP-tagged p53 (p53/EGFP). In the first experiment, cells were lysed twenty-four hours posttransfection. Subsequent coimmunoprecipitation of IL-1α/Flag and p53/EGFP was performed using anti-GFP antibody. Western blot analysis revealed that a fraction of IL-1α coprecipitated with p53 (Fig. 2a,b). Association of transiently expressed IL-1α/Flag and p53/EGFP was further confirmed in the U2OS cell line using GFP-Trap agarose beads (ChromoTek). In the control experiment we also showed that U2OS cells coexpressing EGFP together with IL-1α/Flag did not yield any precipitation of IL-1α/Flag with GFP-Trap beads (Supplementary Fig. 6).

To confirm our observation in a noncancerous cell line, a second reciprocal coimmunoprecipitation experiment was performed using the Mrc-5 normal diploid fibroblast–like cell line. Cells were transiently cotransfected with recombinant plasmids encoding p53/EGFP and Flag–tagged IL-1α, and coimmunoprecipitation was performed with anti-Flag antibody. A reciprocal coimmunoprecipitation experiment confirmed the interaction of IL-1α with p53 and/or their presence within the same supramolecular complex (Fig. 2c).

### *In situ* PLAs confirmed the close proximity of endogenous IL-1α and p53 within the cell

**Figure 3:**
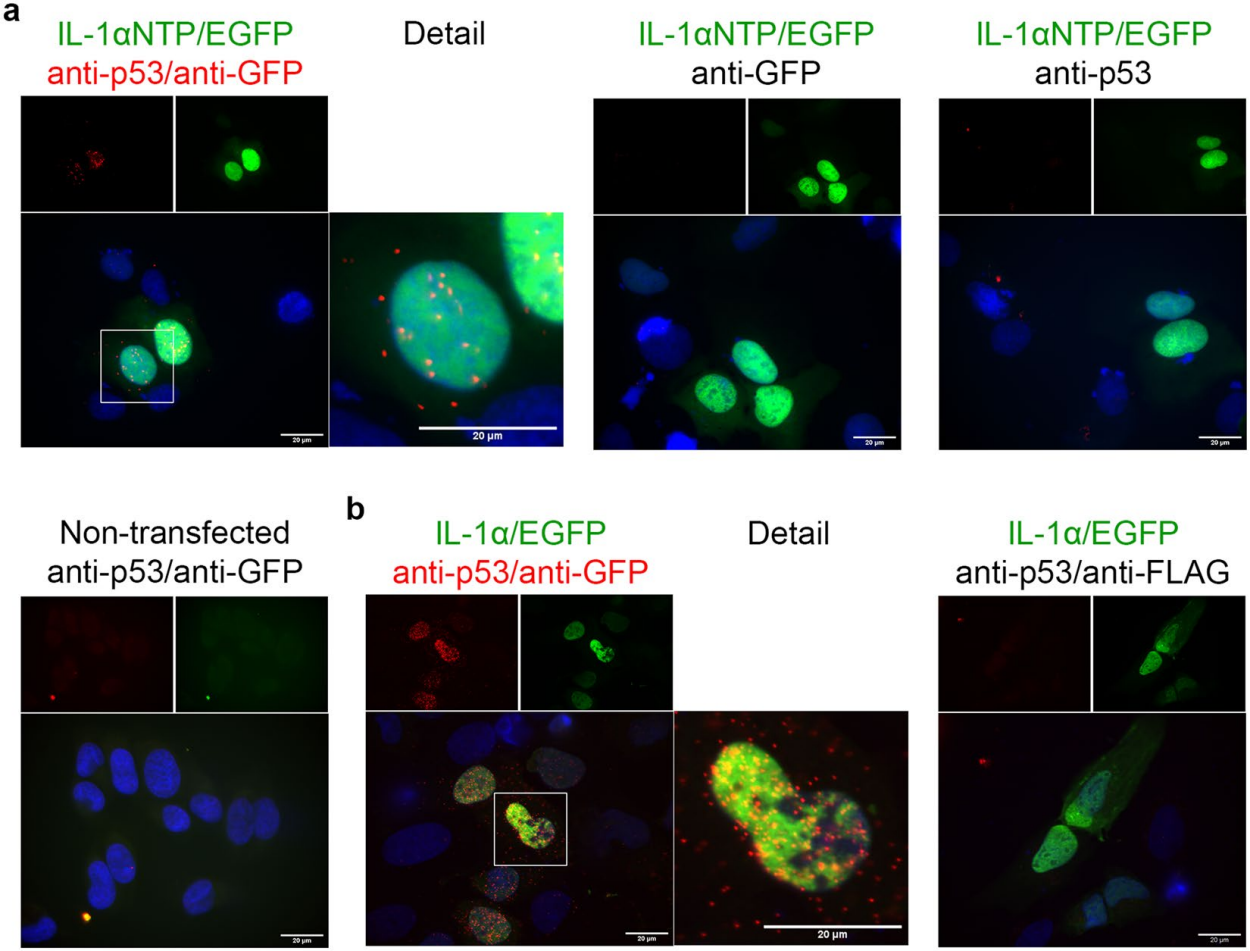
*In situ* proximity ligation assay (PLA) indicates the close colocalization of p53 with IL-1αNTP and IL-1α in U2OS cells. (**a**) U2OS were transfected with expression plasmid encoding fusion IL-1αNTP/EGFP protein (green captions and signals). PLA foci (red) developed both in nuclei and in the cytoplasm of transfected cells treated simultaneously with anti-GFP and anti-p53 (CM-1) antibodies. PLAs in transfected cells treated with single antibody or in untransfected cells treated with anti-GFP and anti-p53 together did not develop any signal and served as negative controls. (**b**) PLA with anti-p53 and anti-GFP antibodies was performed in U2OS cells which were transfected with expression plasmid encoding the full-length IL-1α/EGFP fusion protein and then treated with 20 μM roscovitine for 24 hours. Similarly to IL-1αNTP, the putative full-length IL-1α- and p53-containing complexes were distributed across nuclei and the cytoplasm. PLA in transfected cells using simultaneous application of anti-FLAG and anti-p53 was used as another negative control for both experiments depicted in (a,b) panels. Images were acquired with an Olympus Cell-R wide-field microscope. Scale bars represent 20 μm.

Previous structure modeling and functional assays suggested IL-1α NTP as an important domain for IL-1α intracellular function^18,19^. To confirm the possible close association of IL-1α and p53 in the cellular environment by a different approach, we employed Duolink technology based on the *in situ* proximity ligation assay (PLA). In this assay, we transfected U2OS cells with expression plasmids coding either for full-length IL-1α or IL-1α NTP, both fused with EGFP. The anticipated interactions between the endogenous p53 protein and transiently produced either IL-1α/EGFP or IL-1αNTP/EGFP were visualized using anti-GFP antibody and rabbit polyclonal anti-p53 antibody CM-1 in the PLA assay. Discrete PLA fluorescence foci indicating colocalization events were found in cell nuclei in both cases. Unexpectedly, however consistent with the results of the previous experiments depicted in Fig. 1, PLA signal was also detected in the cell cytoplasm for both p53•IL-1α and p53•IL-1α-NTP protein pairs, thus indicating the role of IL-1α NTP in the possible mutual association of p53 and IL-1α proteins. Separate control experiments with single anti-GFP antibody or anti-p53 antibody did not produce any PLA signal. Similarly, application of both antibodies in untransfected cells or application of anti-Flag antibody instead of anti-GFP antibody did not lead to development of any PLA foci. (Fig. 3)

**Figure 4:**
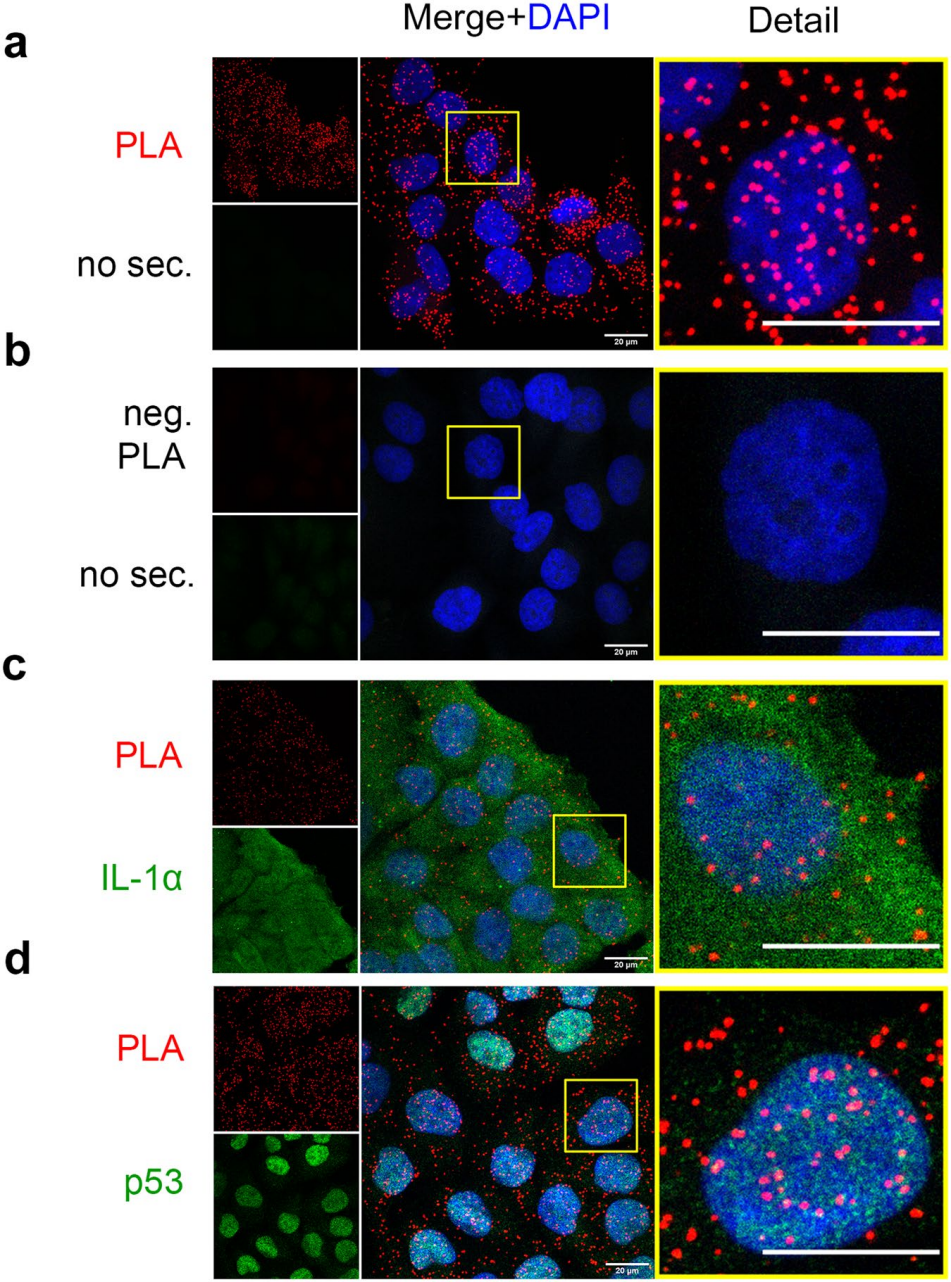
*In situ* proximity ligation assay (PLA) indicates the close colocalization of endogenous IL-1α and p53 in A375 cells. The localization of either IL-1α or p53 (both in green) was detected using indirect immunofluorescence microscopy together with PLA, which revealed foci in which p53 and IL-1α appeared in close proximity to each other (red). (**a**) The interaction of IL-1α and p53 was visualized by PLA without the additional staining of endogenous proteins; (**b**) negative PLA control using anti-GFP antibody instead of anti-IL-1α antibody without the additional staining of endogenous proteins; (**c**) PLA with the staining of endogenous IL-1α. The nucleocytoplasmic distribution of both IL-1α and the PLA signal is clearly visible. (**d**) PLA was performed with staining for endogenous p53. The majority of the p53 protein localized to cell nuclei; however, a fraction of p53 was still present in the cytoplasm. PLA signal documenting the interaction between p53 and IL-1α is present in both the nucleus and cytoplasm. Images were captured with a Zeiss LSM 880 confocal microscope, and scale bars represent 20 µm. “no sec”. means that no secondary antibody was added to simultaneously label either endogenous p53 or IL-1α.

Then we wanted to test whether we will be able to confirm the positive PLA results also with the endogenous IL-1α and p53 proteins. We decided to use human melanoma A375 cells which are known for their increased constitutive production of IL-1α^34^. As evidenced in Fig. 4a, mouse monoclonal antibody against the IL-1α precursor and rabbit polyclonal anti-p53 antibody CM-1 developed discrete foci in PLA assay which were, in concordance with the previous results, abundant both in nuclei and the cytoplasm. No PLA signal was detected in negative control samples using anti-GFP antibody instead of anti-IL-1α antibody (Fig. 4b).

To further analyze the PLA results in the context of endogenous protein localization, we modified the original PLA protocol. In this setup, we performed the PLA using A375 cells as before but added goat secondary antibodies conjugated with Alexa Fluor 488 recognizing either mouse primary antibody against IL-1α (Fig. 4c) or rabbit primary antibody against p53 (Fig. 4d) during the amplification step of the original protocol. This modification allowed us to simultaneously visualize PLA colocalization events and the distributions of individual endogenous proteins within single cells. Furthermore, even with the relatively low level of staining for endogenous p53 in the cytoplasm, a considerable PLA signal indicating an IL-1α and p53 interaction was present in both the nucleus and cytoplasm (Fig. 4). The PLA signal was essentially evenly distributed between the nucleus and cytoplasm in the experiment depicted in Fig. 4, whereas IL-1α was found across the whole cell, and p53 was found predominantly in the nucleus. Thus, the PLA signal did not follow distribution of the p53 protein within the cell. This result indicates the specificity of the PLA and suggests that only a minor fraction of p53 and IL-1α interact and/or participate in the same supramolecular complex (Fig. 4). Subsequently we found that the PLA assay can readily detect close colocalization of p53 and IL-1α in nuclei and the cytoplasm of HeLa and U2OS cells as well (Supplementary Figs. 1 and 2).

### Etoposide treatment increases the frequency of the IL-1α•p53 interaction in the nucleus

The tumor suppressor p53 is a well-known regulator of the DNA repair machinery. IL-1α was previously shown to be a DNA damage sensor^33^. Therefore, we decided to investigate whether the frequency and intracellular distribution of PLA foci indicating the IL-1α and p53 interaction could be modulated by a DNA-damaging agent. We used etoposide (10 µg/ml) treatment for two hours, as described by Cohen *et al*.^33^, to induce DNA damage in the U2OS osteosarcoma cell line. DMSO solvent had no considerable effect on the PLA signal (Supplementary Fig. 3).

**Figure 5:**
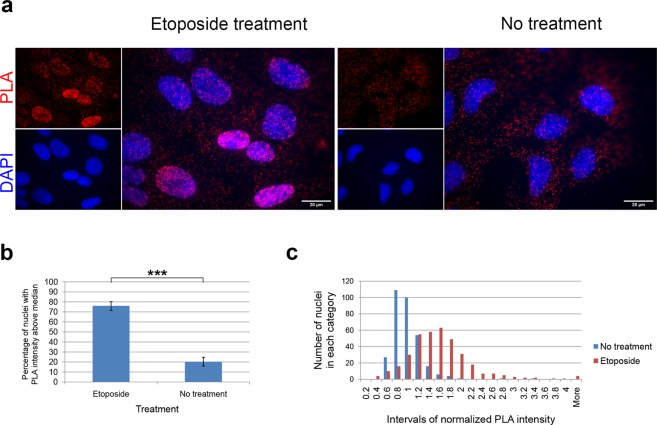
Effect of etoposide treatment on the p53•IL-1α interaction, as detected by *in situ* proximity ligation assay (PLA). PLA was performed with mouse anti-IL-1α and rabbit anti-p53 antibodies in U2OS cells treated with 10 μg/ml etoposide for two hours (n = 364) or control untreated cells (n = 321). (**a**) Representative fluorescence microscopy images used for subsequent analysis of the PLA intensity in nuclei and to compute *I*_n_; etoposide-treated cells show a considerably higher PLA intensity in their nuclei in comparison with untreated cells. (**b**) PLA intensities in the nuclei of cells treated with etoposide were higher than those in untreated cells. The statistical significance of the results was evaluated by Fisher’s exact test (***p < 0.001); error bars represent 95% confidence intervals calculated by the adjusted Wald method. (**c**) Mean PLA fluorescence intensities over all pixels within individual nuclei (*I*_n_) were determined for all cells and normalized to respective median *I*_n_ in the given biological replicate. The numbers of nuclei whose normalized *I*_n_ values fell into discrete intervals were plotted as a histogram. The PLA fluorescence was detected with a wide-field Olympus Cell-R microscope, and the scale bars represent 20 µm. Images were processed in ImageJ with a macro described in the Supplementary methods to determine the mean fluorescence intensity of each nucleus. Data were collected from three independent biological replicates. Primary data and all calculations are present in the Supplementary Data File.

The PLA signal was more intense in cells treated with etoposide, indicating an increase in the frequency of the p53•IL-1α association. Representative PLA-labeled U2OS cells from one of three biological replicates used for subsequent analysis are shown in Fig. 5a. We determined the mean PLA fluorescence intensity over all pixels within the individual nuclei (*I*_n_) of treated (n = 364) and untreated (n = 321) cells. To take into account experimental variability and slight differences in the microscope settings, we computed median *I*_n_ values in each biological replicate combining both etoposide treatment and control conditions and counted nuclei with *I*_n_ higher than median *I*_n_ within each given experiment. Across all three biological replicates, 76.1% of nuclei in etoposide-treated cells showed higher *I*_n_ than median *I*_n_ of the corresponding biological replicate, whereas *I*_n_ exceeded this threshold in only 20.3% of nuclei in untreated control cells (Fig. 5b). To analyze the distribution of combined PLA fluorescence data from all three biological replicates, we normalized *I*_n_ values in each experiment with corresponding median *I*_n_ and counted nuclei with a normalized *I*_n_ that fell into discrete intervals from both treated and untreated cells (Fig. 5c). Normalized *I*_n_ values in etoposide-treated cells did not follow a normal distribution according to the Shapiro-Wilk test; however, the normality of their distribution could not be excluded (p = 0.417598) after the exclusion of outliers with strong signal (n = 14). The distribution of PLA intensities in the nuclei of the untreated population was asymmetrical and right-tailed. Both distributions documented a clear overall increase in the nuclear PLA fluorescence signal (*I*_n_) in the etoposide-treated cells in comparison to the untreated control cells. We observed at least a few p53•IL-1α association events in all cells, regardless of etoposide treatment. Similarly we tested the effect of etoposide treatment on p53•IL-1α-associated PLA foci distribution in the A375 and HeLa cell lines. In agreement with the results obtained with U2OS cells, we found that etoposide treatment induces accumulation of p53•IL-1α-associated PLA signal also in nuclei of HeLa and A375 cells, even though this accumulation was less profound in HeLa cells (Supplementary Figs. 2 and 4).

**Figure 6:**
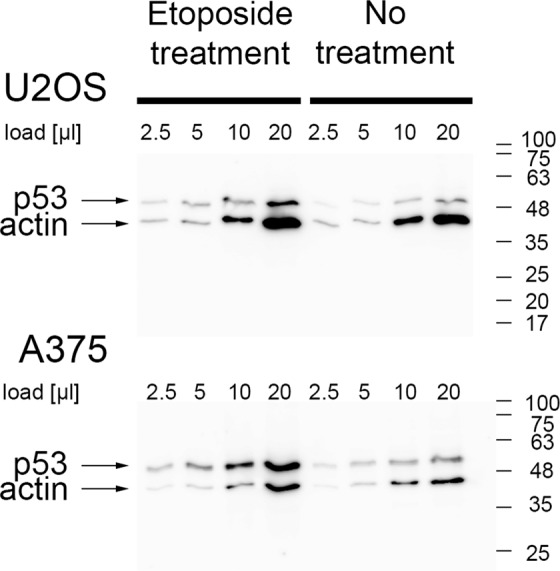
Etoposide treatment increases intracellular levels of the p53 protein in U2OS and A375 cells. Serial dilutions of cell lysates (load) from untreated cells and cells treated with 10 µg/ml etoposide for two hours were analyzed by western blotting. Membranes were developed with anti-β-actin and anti-p53 (DO-1) antibodies. Numbers on the side correspond to the protein MW ladder.

Increase of p53 intracellular levels upon etoposide treatment has been documented in many cells including human embryonic stem cells and the U2OS cell line^9,35,36^. Increased intracellular levels of p53 may account to the increased PLA signal associated with p53•IL-1α complexes. Therefore we wanted to estimate p53 levels in our experimental conditions. We treated A375 and U2OS cells with etoposide (10 µg/ml) for two hours exactly as in the other experiments and analyzed a serial dilution of cell lysates simultaneously for p53 and β-actin using western blotting (Fig. 6). Measurement of p53 signal intensities normalized by signal from β-actin revealed an average increase in p53 protein levels in etoposide–treated cells 1.9 times and 2.3 times in U2OS and A375 cells respectively.

**Figure 7:**
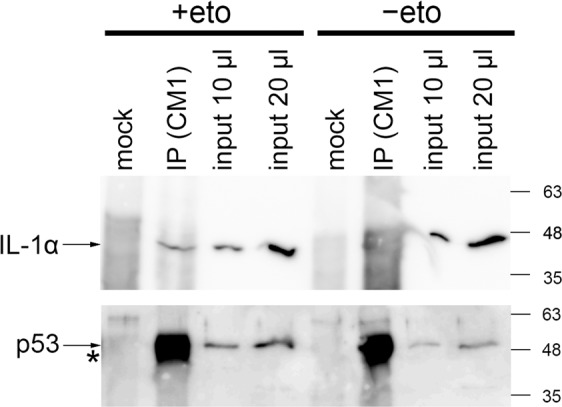
Endogenous IL-1α and p53 coimmunoprecipitate from U2OS cells upon etoposide treatment. Cells were either treated with etoposide (10 µg/ml, 2 hours, +eto) or left untreated (-eto). Coimmunoprecipitation (IP) was performed with anti-p53 antibody (CM-1) and resulting protein samples were split and analysed for IL-1α and p53 content by western blotting in parallel. The IL-1α-specific band is clearly visible in IP from etoposide-treated cells. Asterisk marks an antibody heavy chain. Numbers on the side correspond to the protein MW ladder. Mock IPs were performed from the same amount of the corresponding lysates without adding the CM-1 antibody. For the full-size membrane records we refer to the Supplementary Fig. 7a,b. Similar results we obtained also from A375 cells (Supplementary Fig. 7c). Supplementary Fig. 8 documents efficient imunoprecipitation of the p53 protein from the lysates.

Initially we experienced difficulties with immunoprecipitation of p53•IL-1α complexes formed by endogenous proteins. We thus wanted to test if the observed changes in subcellular localization of p53•IL-1α-associated PLA foci and their abundance upon etoposide treatment might improve the detection of these complexes by means of immunoprecipitation. Immunoprecipitation and the subsequent detection of endogenous p53 is complicated by its molecular weight similar to the antibody heavy chain. Therefore we used p53 protein as a bait. An experiment with U2OS cells showed that while the endogenous IL-1α was barely visible in the fraction bound to anti-p53 antibody (IP) in untreated cells, we were able to detect a specific IL-1α signal in the IP fraction of etoposide–treated cells (Fig. 7).

**Figure 8:**
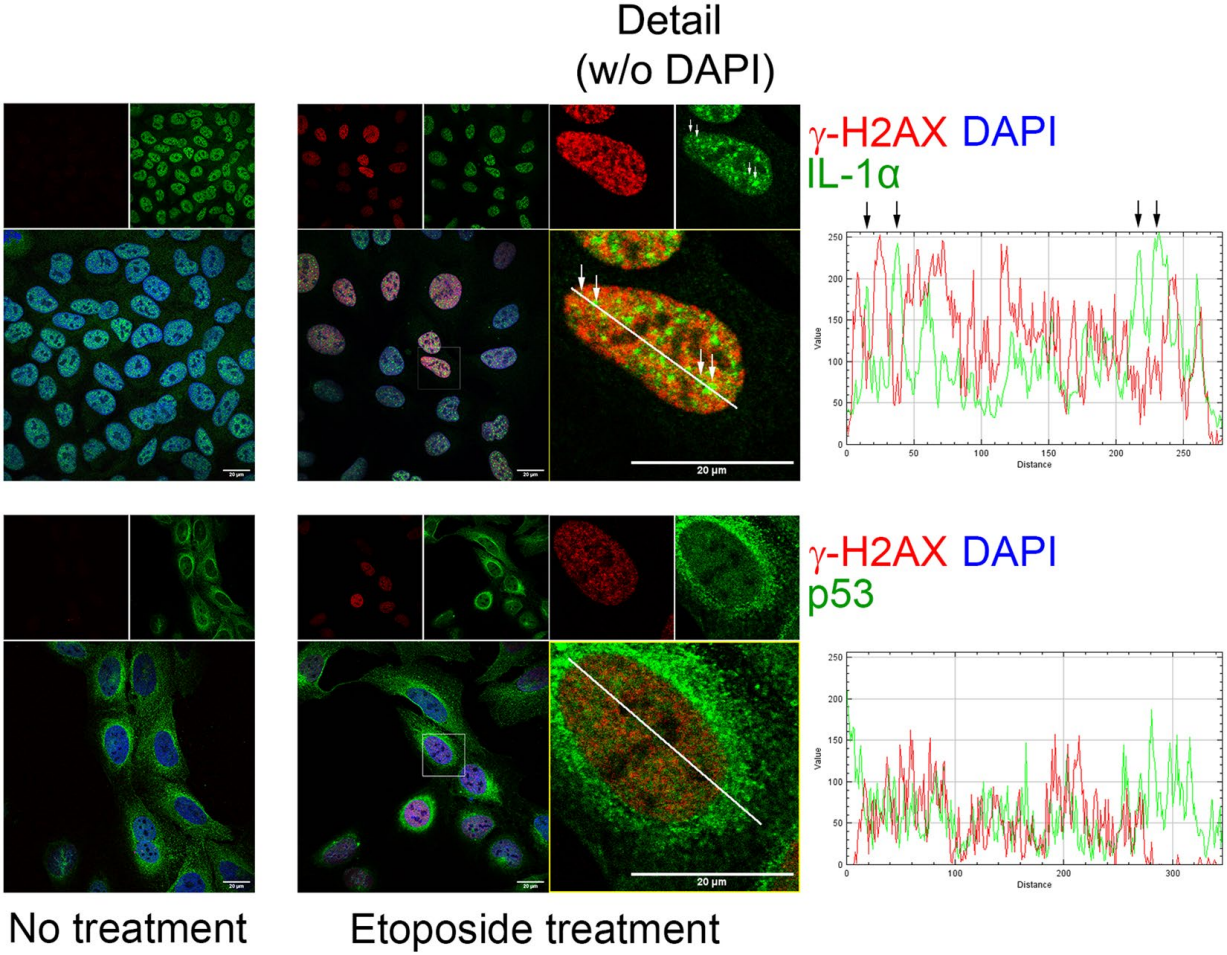
Etoposide did not induce colocalization of IL-1α and γ-H2AX in the nucleus of U2OS cells. Cells were treated with etoposide (10 µg/ml, 2 hours) or left untreated. Upper panels show staining for endogenous γ-H2AX (red) and IL-1α (green); lower panels demonstrate staining for endogenous γ-H2AX (red) and p53 (green). Nuclei were stained with DAPI. The intensity profiles show the fluorescence intensity of both labeled proteins in the regions delineated by white lines. Etoposide increases relocalization of IL-1α into cell nuclei and induces formation of both IL-1α and γ-H2AX foci. Arrows above the white line in the γ–H2AX and IL-1α merge image and above the corresponding intensity profile show larger IL-1α foci depleted of the γ-H2AX signal. Images were captured with a Leica SP8 confocal microscope. Scale bars represent 20 µm.

Etoposide is a compound commonly used to induce focal accumulation of γ-H2AX in the nucleus at sites of DNA damage^37^. U2OS cells readily developed γ-H2AX foci upon treatment with 10 µg/ml etoposide for two hours. We haven’t seen any sign of IL-1α and γ-H2AX colocalization. We could even observe dramatic decrease of γ-H2AX signal in those sites where IL-1α accumulated into the large nuclear foci. Contrary to that, an overall view on the intensity profiles reveals that nuclear regions showing increased p53 accumulation appear to demonstrate increased signal specific to γ-H2AX as well. However, due to the large amount of both proteins in the nucleus of etoposide–treated cells it is difficult to clearly distinguish if there is any close colocalization of these two proteins, even though certain peaks from the profile could suggest that. We also cannot rule out an interaction between a minor fraction of IL-1α and γ-H2AX outside larger and clearly defined nuclear foci of both proteins (Fig. 8).

## Discussion

In our previous studies, we focused on the nuclear function of IL-1α, which has not yet been sufficiently investigated. We provided evidence that IL-1α binds the human histone acetyltransferase complexes p300, PCAF and GCN5^18^. Subsequently, we identified the IL-1α-binding site on the evolutionarily conserved catalytic domain of the STAGA complex using a yeast model^19^. Given that an earlier study by Cohen *et al*. established a link between IL-1α and DNA damage^33^, we aimed to examine the potential association of IL-1α with the tumor suppressor p53, which is also a prominent player in the DNA damage response.

Nuclear localization signals for the p53^1^ and IL-1α^14^ proteins have been previously reported. As expected, both IL-1α and p53 were present in cell nuclei and cytoplasm in our experiments but mostly absent from nucleoli. IL-1α and p53 exhibited moderate colocalization within the cells, although we did not observe any specific nuclear and/or cytoplasmic foci enriched in both investigated proteins.

For the majority of our experiments, we used the A375 human melanoma cell line, which produces the IL-1α precursor in sufficiently high quantities and has been widely used for the study of IL-1α^34,38^, and U2OS cells, which proved suitable for microscopy studies and PLA analysis. For coimmunoprecipitation experiments, we also employed Mrc-5 normal human lung fibroblasts as an example of noncancerous senescent cells. We observed the coimmunoprecipitation of a fraction of transiently expressed IL-1α/Flag and p53/EGFP, regardless of which of these proteins was used as bait. We were also able to coimmunoprecipitate endogenous IL-1α and p53 from U2OS and A375 cells treated with the DNA-damaging compound etoposide. Increased ability of endogenous IL-1α to coprecipitate with p53 upon etoposide treatment might be caused both by the increased levels of either of the interacting partners as well as by the increased stability of the p53•IL-1α-containing complex. Indeed, we and many others have shown that etoposide increases intracellular p53 levels^9,35,36^.

To obtain more accurate information about possible IL-1α and p53 colocalization, we employed the PLA technique, which is currently widely used for protein-protein interaction studies. In theory, the PLA can detect proteins separated by a maximal distance of 30–40 nm^39^. This experimental approach was previously used to study the interaction partners of both p53^40–45^ and IL-1α^46^. The advantage of the PLA is that it can be used to detect interactions between low-abundance endogenous proteins and allows visualization of the sites of these interactions. The PLA is very sensitive and can, in principle, detect single pairs of individual interacting molecules.

Using the PLA, we observed the interaction of endogenous IL-1α and p53 in not only the nucleus but also the cytoplasm of all the tested cell lines. The PLA visualizes only the protein-protein interaction sites and cannot show the overall distribution of individual proteins from the pairs of interacting partners. Therefore, we modified the PLA assay and included individual p53 and IL-1α labeling by indirect immunofluorescence. To avoid a possible decrease in PLA sensitivity due to competition of secondary antibodies with probes holding concatemers, which were created in a course of PLA process, we did not attempt to detect both proteins from the pair together with PLA signal in a single sample.

We also employed the PLA to characterize the effect of etoposide on the IL-1α•p53 interaction. Etoposide was previously shown to promote recruitment of the EGFP/IL-1α fusion protein to DNA damage sites^33^. Our PLA analysis revealed that interacting IL-1α and p53 were scattered across the nuclei and cytoplasm of both control and etoposide-treated cells. However, the abundance of nuclear PLA-labeled foci was remarkably increased upon etoposide treatment. Confocal microscopy images showed the predominant nuclear localization of p53 with a small amount of signal for p53 in the cytoplasm. The observed distribution of PLA foci thus did not reflect a predominant subcellular distribution of p53 in untreated cells. This finding is consistent with the relatively weak coimmunoprecipitation efficiency and modest Pearson’s colocalization coefficient, suggesting that only a small portion of the total cellular p53 was associated with IL-1α in cells under the tested conditions. We obtained similar results from the PLA experiments in all three tested cell lines (U2OS human osteosarcoma cell line, A375 human malignant melanoma cell line and HeLa human cervical cancer cell line). However, the enrichment of nuclei with the PLA signal in etoposide-treated cells was less profound in HeLa cells, even though p53 and IL-1α increased in the HeLa nuclei upon etoposide treatment similarly as in the other two cell lines. This result might be explained by the presence of the integrated HPV-18 viral genome and associated expression of the viral oncoprotein E6^47^ which has been shown to bind to p53^48^ and to interfere with p53 function by promoting its degradation^49^, inhibiting p53 phosphorylation^50^ and preventing its acetylation^51^.

The methods used in our study, coimmunoprecipitation and the PLA, did not allow us to specify whether the observed interaction is direct or indirect. The histone acetyltransferases STAGA and p300 are both known to interact with IL-1α^18,19,52^ and p53; the p53 protein binds four different domains of p300^53^, two of which, the TAZ2 and IBiD domains, are localized in the region of p300, which we previously showed to be required for IL-1α binding^18^. In addition, the p53 protein binds the TAF9, GCN5 and ADA2b subunits of the STAGA complex^54^. Therefore, if the observed IL-1α•p53 interaction is indirect, HAT complexes are candidate scaffolds for the association of these proteins. This idea could be supported by only a moderate increase of p53•IL-1α PLA accumulation in HeLa nuclei upon the etoposide treatment which might account for the E6-mediated inhibition of p53 acetylation by the histone acetyltransferase p300^51^. We also show that ectopically expressed IL-1α NTP possesses an intrinsic ability to generate the PLA signal with endogenous p53. This is in agreement with our previous results showing that NTP is the functional domain mediating interaction of the IL-1α precursor with the histone acetyltransferase complexes^18,19^.

The effect of p53 acetylation by histone acetyltransferases on the function of p53 is complex and depends on the context of other p53 modifications^55,56^. In response to DNA damage, p53 is activated by acetylation by PCAF and p300^57,58^. Hyperacetylation of the C-terminal region of p53 by the p300 protein, however, leads to p53 accumulation in the cytoplasm^59^. The acetylation of p53 changes its affinity for the promoters of its target genes; for example, the acetylation of p53 at K320 leads it to bind only strong p53 promoters, thus increasing cell survival after DNA damage^60^. In the case of IL-1α, acetylation of its nuclear localization sequence was observed to lead to the cytoplasmic accumulation of IL-1α^33^. Despite their name, histone acetyltransferases are common in the cytoplasm, where they contribute to modifying nonhistone substrates, which often changes the localization of such proteins^61^.

We speculate that histone acetyltransferases mediate the increased number of IL-1α•p53 interaction foci observed after etoposide treatment. The importance of the STAGA complex in the DNA damage response is well established, especially after UV damage^62^. The GCN5 histone acetyltransferase is responsible for the acetylation of histone H3 on γ-H2AX-positive nucleosomes and thus contributes to activating the DNA repair machinery^63^. Moreover, such acetylation is also required for the induction of robust H2AX phosphorylation^64^. Recently, the role of the deubiquitination module of the SAGA complex in the induction of γ-H2AX was described^65^. Finally, p300 was found to also play a role in the repair of double-strand DNA breaks (DSBs)^66^.

In our experiments, we observed an increase in PLA colocalization events in cell nuclei after treatment with etoposide, a compound commonly used to induce DSBs and the phosphorylation of H2AX^37^ that was also reported to promote recruitment of the EGFP/IL-1α fusion protein to DNA damage sites^33^. However, we could not observe any recognizable foci or spots formed by endogenous IL-1α and γ-H2AX both in untreated and etoposide-treated U2OS cells (Fig. 8). Despite this, we still cannot rule out the interaction between a minor fraction of IL-1α and γ-H2AX outside the clearly defined nuclear foci. There may be several explanations for this finding. Although etoposide acts as a topoisomerase II poison, some works claim that etoposide activity often results in single-strand breaks and/or resulting DSBs do not trigger the DSB response because the religation of both strands is independently inhibited by etoposide and/or stalled topoisomerase II complexes are not efficiently recognized by ATM and other members of the DSB response machinery. γ-H2AX, an established DSB marker^67^, can thus bind the minority of etoposide-damaged sites only^68^. However, the presence of γ-H2AX foci does not necessarily indicate the presence of DSBs^69^. The infrequent colocalization of p53 and γ-H2AX at DSBs induced by irradiation was reported but has not yet been widely studied^70^. Therefore, we cannot rule out the possibility that the IL-1α•p53 interaction does not occur at etoposide-induced DSBs. Finally, differences in the reported occurrence of γ-H2AX•IL-1α foci might be caused by differences in the cell lines used, high intracellular levels of the transiently produced proteins, usage of endogenous IL-1α versus EGFP-tagged IL-1α which was used in the previous experiments^33^ and/or other variations in experimental parameters. Differences in experimental conditions, protein overproduction and various fusion tags have often been shown to have a remarkable effect on the assembly of various protein-protein and protein-nucleic acid complexes, which has been deeply studied, e.g., in the case of stress granules and processing bodies^71^. Etoposide can also provoke cellular responses in additional ways beyond topoisomerase II poisoning; etoposide can induce the production of reactive oxygen species^72^ and/or change the secondary structure of histone H1^73^.

p53 and IL-1α may encounter each other in various other cellular processes; thus, our observation of their increased association upon etoposide treatment might be only remotely related to etoposide-induced genotoxic stress. p53 proapoptotic activity can be blocked by inflammation-induced NF-κB activation, and in contrast, p53 can block NF-κB-driven inflammation, as described extensively elsewhere^74,75^. p53 and NF-κB also compete for the limited transcription factor p300 and its homolog, CREB-binding protein^76,77^. Blockade of the proinflammatory IL-1α signaling pathway was suggested as a candidate therapeutic target in cancer to restore p53 activity^78^. Intracellular IL-1α was indeed shown to be a transforming oncoprotein in glomerular mesangial cells^79^ and to promote proliferation via NF-κB activation in head and neck squamous cell carcinomas^80^ and T-lymphocytic leukemia cells^24^. However, IL-1α was also shown to induce apoptosis^17^ or block proliferation^81^ in some tumor cells.

We have described a novel interaction between the dual-function cytokine IL-1α and the tumor suppressor p53 using fluorescence microscopy, coimmunoprecipitation and PLA. While IL-1α was previously identified among genes preferentially required for the proliferation of p53-deficient human cancer cell lines^82^, our study is the first to show the colocalization of those proteins in a putative macromolecular complex. We have also shown that the observed interaction between IL-1α and p53 can be modulated using the antineoplastic drug etoposide, suggesting a possible role for the IL-1α•p53 interaction in DNA repair, in accordance with a previously published finding that IL-1α functions as a DNA damage sensor^33^.

## Methods

### Cells and treatment

The A375 human malignant melanoma cell line, Mrc-5 normal fibroblast-like cell line, U2OS osteosarcoma cell line and HeLa cervical cancer cell line were cultivated in a humid atmosphere at 5% CO_2_ in DMEM (Gibco) supplemented with 10% FBS and 2 mM L-glutamine. To induce DNA damage, cells were treated with 10 µg/ml etoposide (Selleckchem, S1225) for two hours. Working concentration of etoposide was diluted from 50 mg/ml stock in DMSO (Sigma-Aldrich, D2650)

### Fluorescence microscopy

Cells grown on round ⌀12 mm microscope coverglasses were fixed with 4% PFA in PBS for 20 min at room temperature, permeabilized with 0.5% Triton X-100 for 5 min, washed 3× with PBS, blocked with 0.25% BSA and 0.25% gelatin in PBS and incubated for 1 hour at room temperature with mouse monoclonal antibodies against IL-1α diluted 1:100 (I7409, Sigma), rabbit polyclonal anti-p53 antibody CM-1 diluted 1:100 (kindly provided by Bořivoj Vojtěšek), monoclonal anti-p53 antibody diluted 1:100 (Chemicon, MAB4040) and rabbit monoclonal anti-γ-H2AX diluted 1:500 (ab81299, Abcam). The cells were then washed 3 × 5 min in PBS and incubated with Alexa Fluor 594 goat anti-mouse and Alexa Fluor 488 goat anti-rabbit (Invitrogen) diluted 1:1000. The cells were then washed for 5 min once with PBS, once with PBS containing DAPI (0.1 μg/ml), once with PBS and briefly in deionized water. Samples were then mounted in ProLong Gold antifade reagent (Life Technologies).

Images for quantification were captured using the Cell-R system on an Olympus IX81 inverted microscope with a UPLSAPO 60× objective, DAPI/Texas Red filters (83000v2), U-MWIBA3 GFP filter and a Hamamatsu ORCA C4742-80-12AG camera. Images were also captured with confocal microscopes Zeiss LSM 880 equipped with a PlanApochromat 63×/1.4 Oil DIC M27 objective with detectors set to 410–480 nm, 481–544 nm or 552–641 nm and a 405 diode laser, argon laser or DPSS 561 laser respectively, and Leica SP8 equipped with HC PL APO CS2 63×/1.40 OIL objective and hybrid detector set to 493 nm-580 nm and PMT detector set to 581 nm-751 nm together with 405 diode laser or argon laser and DPSS 561 laser respectively.

### *In situ* PLA

For the *in situ* PLA, we used the Duolink *in situ* orange kit (Sigma-Aldrich, DUO92007) according to the manufacturer’s recommendation using solutions supplied in the kit. Cells were grown on round microscope coverglasses (⌀12 mm), fixed with 4% PFA in PBS for 20 min at room temperature and permeabilized with 0.5% Triton X-100 for 5 min. Cells were blocked with blocking solution for 30 min at room temperature and subsequently incubated with rabbit polyclonal anti-p53 antibody CM-1 and mouse monoclonal anti-IL-1α antibody I7409 (Sigma-Aldrich) or mouse monoclonal anti-GFP antibody (B2, Santa Cruz Biotechnology) as a negative control for 1 hour at 37 °C. Alternatively, U2OS cells were transfected with expression plasmids coding for either IL-1α/EGFP or IL-1αNTP/EGFP using the TurboFect transfection reagent (Thermo Scientific). PLA in transfected cells was performed with anti-GFP and CM-1 anti-p53 antibodies. Control experiments were performed following manufacturer’s recommendation using either individual antibodies or anti-p53 antibody in combination with anti-FLAG antibody (Sigma-Aldrich, F1804) instead of anti-GFP antibody. All antibodies were diluted 1:100 in the blocking solution supplied in the kit. After the PLA, the cells were washed for 5 min once with PBS, once with PBS containing DAPI (0.1 μg/ml), once with PBS, and briefly in deionized water and mounted in ProLong Gold antifade reagent (Life Technologies). To visualize endogenous proteins, we performed PLA as described and in addition, goat anti-rabbit or anti-mouse antibodies conjugated with Alexa Fluor 488 (Life Technologies) diluted 1:1000 were added during the amplification step of the PLA protocol.

### Image and statistical analysis

Images later subjected to statistical analysis were captured on an Olympus Cell-R microscope using an UPLSAPO 60× objective under the same conditions in each experiment and processed with the Fiji 1.52 (ImageJ2) software^83,84^ to determine the mean PLA signal intensity in each nucleus; the ImageJ macro used for batch processing is available in the Supplementary Methods. For each experiment, the median nuclear PLA intensity was determined, and the number of nuclei with the PLA signal intensity above the median was computed. The average nuclear PLA signal intensity across three biological replicates was determined as a maximum likelihood estimate, and the 95% confidence interval was determined using the adjusted Wald method. Statistical significance was examined by Fisher’s exact test. The distribution of the data was tested using the Shapiro-Wilk test and visualized as a histogram according to consecutive, nonoverlapping and equal increments of 0.2. The data from image and statistical analyses are available in Supplementary Data.

### DNA constructs and transient transfection

The plasmid encoding Flag-tagged full-length IL-1α was described previously^18^, and the p53/EGFP expression vector was constructed by J. Stommel^85^. For transient production of IL-1αNTP/EGFP and IL-1α/EGFP fusion proteins in human cell lines, both full-length IL-1α and IL-1αNTP(1–112 AA) coding regions were inserted into the pEGFP-C1 expression vector, which was also used alone as a control. For transient expression of the vectors in A375, U2OS and Mrc-5 cells, TurboFect transfection reagent (Thermo Scientific) was used according to the manufacturer’s instructions. Transfection efficiency varied between 20% and 40%, depending on the cell line.

### Western blotting

The method was performed essentially as described previously^19,86^. Proteins separated on 12% SDS-PAGE gels were electroblotted onto nitrocellulose membranes (NC2; Serva). The membranes were blocked in 5% skimmed milk in a TBS-Tween blocking solution (50 mM Tris-HCl, pH 7.5, 150 mM NaCl and 0.5% Tween-20). The following antibodies were used: mouse monoclonal anti-Flag M2 antibody (Sigma-Aldrich, F1804, 1:1000), monoclonal anti-p53 antibodies (Chemicon, MAB4040 and DO-1, 1:1000), rabbit polyclonal anti-p53 antibody CM-1 (1:1000), anti-β-actin antibody (Sigma-Aldrich, A2228, 1:5000), mouse monoclonal anti-IL-1α antibody I7409 (Sigma-Aldrich, 1:1000) and anti-GFP antibody (SantaCruz,sc-9996, 1:1000). Membranes were incubated in blocking solution containing diluted primary antibody at 4 °C overnight. After washing in TBS-Tween and subsequent incubation in blocking solution for 1 hour, the membranes were incubated with either of the corresponding secondary antibodies diluted 1:3000: goat anti-mouse HRP-conjugated antibody (Santa Cruz Biotechnology, sc-516102), swine anti-rabbit HRP-conjugated antibody (Sevapharma) or goat anti-rabbit IgG (H + L)-HRP conjugate (Bio-Rad, 170-6515). Finally, the membranes were immersed in a luminol detection solution and exposed to X-ray film (Medix; FOMA) or directly analyzed with the ImageQuant LAS-4000 imager. If needed, membranes were stripped in stripping buffer (62 mM Tris-Cl, pH 6.8, 2.4% SDS, 106 μM β-mercaptoethanol) at 50 °C twice for 30 min, washed extensively in distilled water and then in TBS-Tween, incubated in blocking solution for 1 hour and processed with additional antibody.

### Coimmunoprecipitation from mammalian cells

The method was performed essentially as described previously^86^. Transiently transfected cells grown in 6-cm culture dishes were washed with PBS and 1 ml of IP lysis buffer (50 mM Tris-Cl, pH 7.6, 150 mM NaCl, 1.5 mM MgCl_2_, 1 mM EDTA, pH 8, 0.5% NP-40, 10% glycerol) supplemented with cOmplete Mini EDTA-free protease inhibitor cocktail (Roche) and 1 mM PMSF. Cells were scraped into an Eppendorf tube, incubated on ice for 30 min and vortexed repeatedly. Samples were centrifuged for 10 min at 13000 × g at 4 °C, and supernatants were transferred into new tubes and kept on ice. Then, 100 μl of buffer B (20 mM HEPES, pH 8, 25% glycerol, 420 mM NaCl, 1.5 mM MgCl_2_, 0.2 mM EDTA, pH 8) supplemented with protease inhibitor cocktail (cOmplete, Roche) and 100 μM DTT was added to the pellet, vortexed and incubated on ice for 15 min. After a 10 min centrifugation at 13000 × g at 4 °C, the buffer B supernatant was added to lysate obtained with IP lysis buffer. For preclearing, 50 μl of protein G-agarose slurry was added to the lysate and incubated for 1 hour at 4 °C while rotating. The tubes were centrifuged at 2000 × g for 20 sec, and the lysates were transferred into a new tube. Then, 70 μl of protein G-agarose slurry and 1 μg of mouse monoclonal anti-Flag antibody (Sigma-Aldrich, F1804) or anti-GFP antibody (B2; Santa Cruz Biotechnology) was added to the lysates, and samples were incubated overnight while rotating at 4 °C. The next day, protein G-agarose slurry containing bound proteins was washed 3 × 15 min with ice-cold IP lysis buffer and centrifuged at 2000 × g for 20 sec, 60 μl of 2 × SLB (0.1 M Tris-HCl, pH 6.8, 20% glycerol, 2% β-mercaptoethanol, 4% SDS and 0.04% bromophenol blue) was added, and the sample was incubated at 100 °C for 5 min. 10–20 µl of either lysate (input) or immunoprecipitated proteins (IP) were separated in SDS-PAGE gels and analysed by western blotting method. Depending on the cell line, the 10 µl of input and IP roughly corresponded to proteins from 3 ×10^4^ and 5 ×10^5^ cells, respectively.

For immunoprecipitation of endogenous proteins, samples were prepared essentially as those from the transfected cell lines, except that the entry amount of cells was scaled up to four 10-cm culture dishes with cells at 80% confluency for the each sample. Protein complexes were precipitated with anti-p53 antibody (CM-1). In this case, thus 10 µl load of both input and IP roughly corresponded to 7 × 10^4^ and 4 × 10^6^ cells, respectively.

## Supplementary information

Supplementary Information.

## Data Availability

The datasets generated and analyzed in the current study are available in the Supplementary information and from the corresponding author on reasonable request.
