# Supplementary material for: Interleukin-1α associates with the tumor suppressor p53 following DNA damage
Source: Sci Rep. 2020 Apr 24;10:6995. doi: 10.1038/s41598-020-63779-x (PMC7181607; doi:10.1038/s41598-020-63779-x)
Supplement: Supplementary file 1 — Supplementary Information. [file 41598_2020_63779_MOESM1_ESM.pdf]

Supplementary information

Interleukin-1α associates with the tumor suppressor p53 following DNA damage

Novak J, Zamostna B, Vopalensky V, Buryskova M, Burysek L, Doleckova D. and Pospisek M*

*Correspondence to: martin.pospisek@natur.cuni.cz

Supplementary figures:

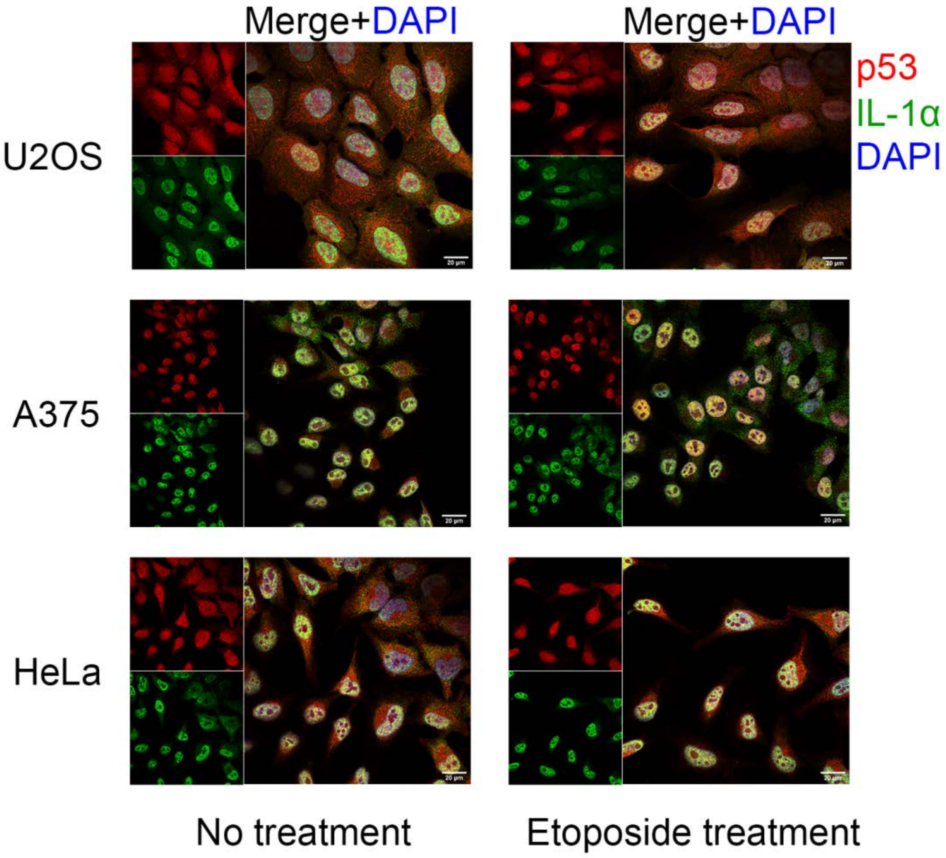

Supplementary figure 1: Subcellular localization of IL-1α and p53 in the U2OS human osteosarcoma cell line, A375 human malignant melanoma cell line and HeLa human cervical cancer cell line. Endogenous IL-1α (green) and p53 (red) were labeled using indirect immunofluorescence. Nuclei were visualized by DAPI (blue, shown in merge). Cells were either treated with etoposide (10 µg/ml, two hours) or left untreated. Images were captured with a Leica SP8 confocal microscope. Both IL-1α and p53 localize mostly to the cell nucleus, with some signal present in the cytoplasm as well. Scale bars represent 20 µm.

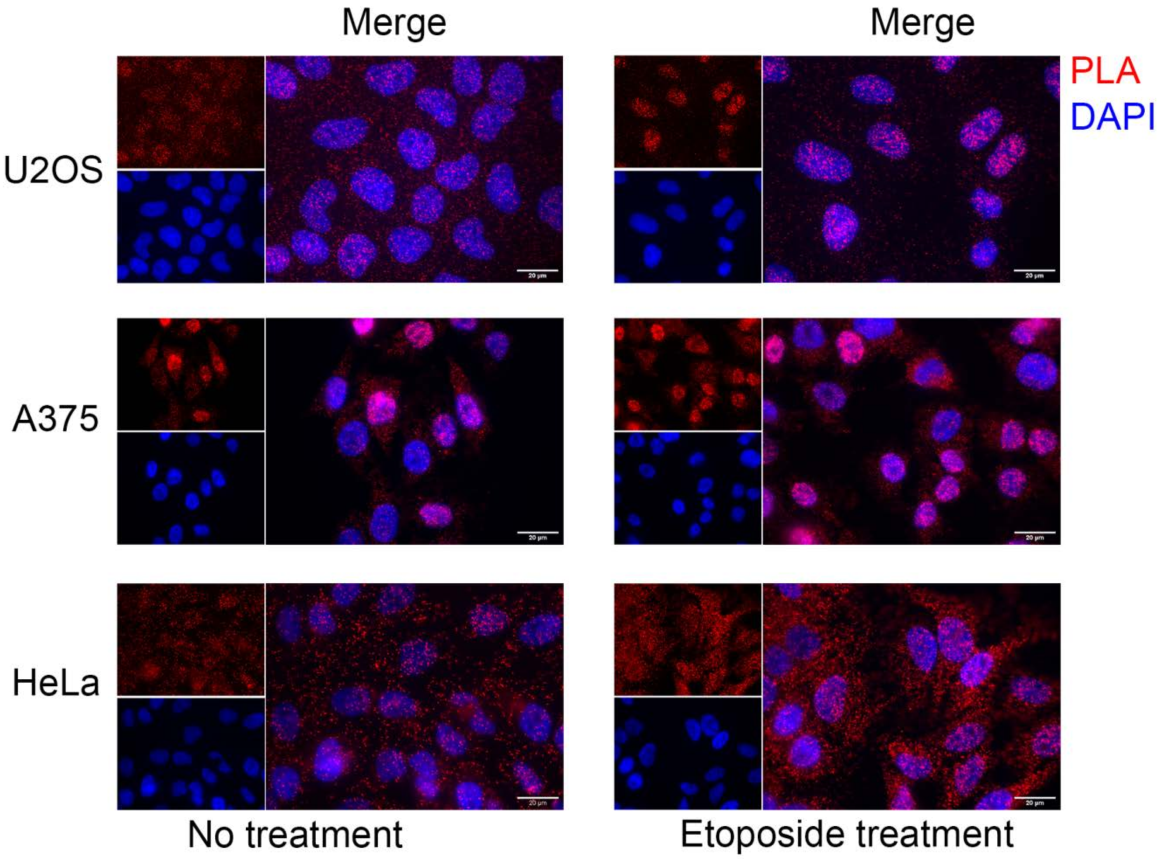

Supplementary figure 2: In situ proximity ligation assay (PLA) indicates the close colocalization of endogenous IL-1α and p53 in U2OS, A375 and HeLa cells. PLA foci (red) were developed with mouse monoclonal anti-IL-1α and rabbit polyclonal anti-53 (CM-1) antibodies. All cells showed the PLA signal distributed across the cytoplasm and nuclei whereas only U2OS and A375 cells displayed a substantial increase in nuclear PLA fluorescence upon treatment with etoposide (10 µg/ml) for two hours. HeLa cells showed remarkable increase in PLA signal upon the etoposide treatment as well however the PLA foci remained evenly distributed across the cells. Nuclei were stained with DAPI (blue). Fluorescence was detected with an Olympus Cell-R wide-field microscope; scale bars represent 20 µm.

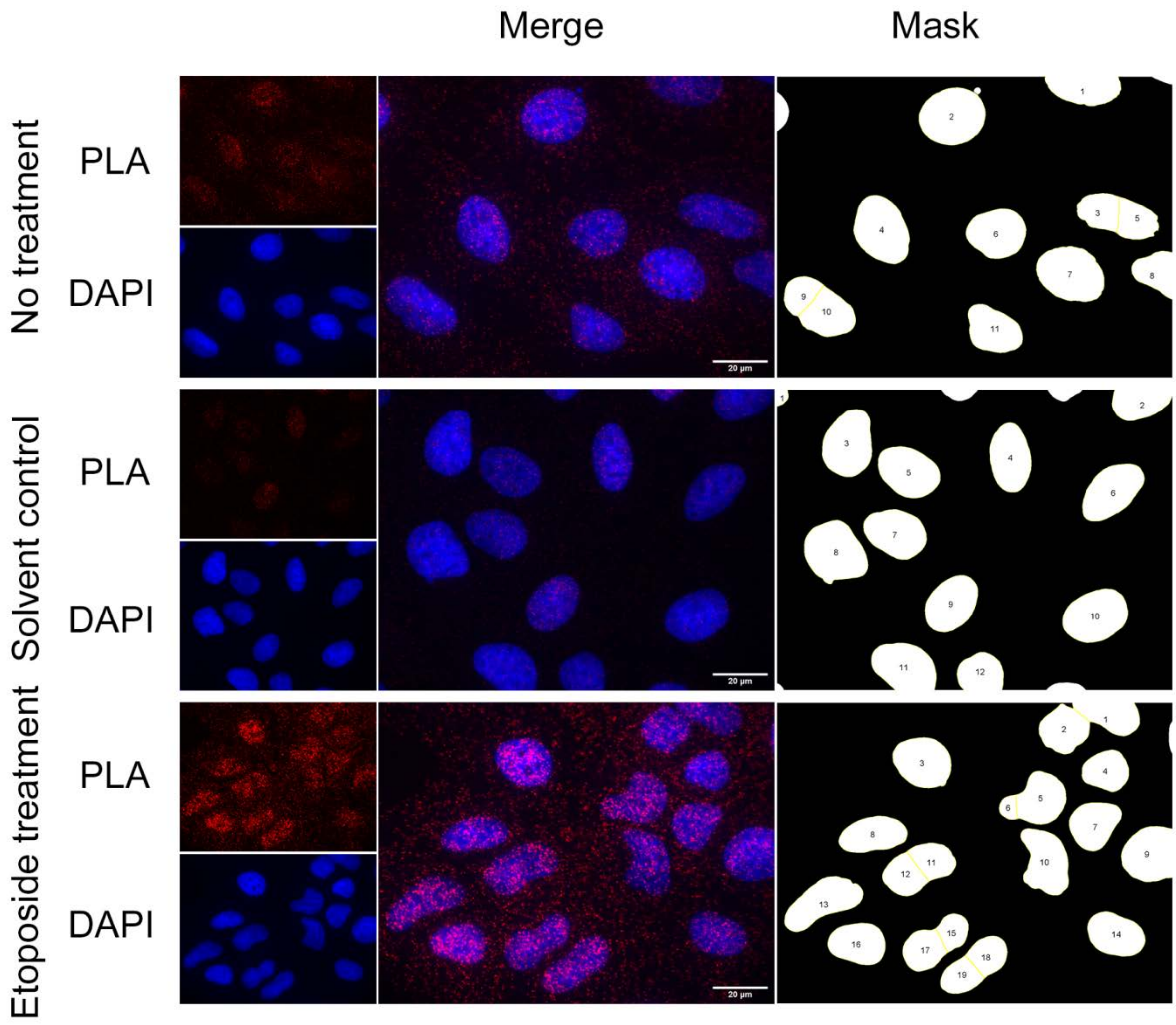

Supplementary figure 3: Solvent control and the selection of nuclei in the *in situ* proximity ligation assay. The *in situ* proximity ligation assay (red) using mouse anti-IL-1α and rabbit anti-p53 antibodies was performed with untreated U2OS cells and U2OS cells treated with 0.02% DMSO (solvent control) or etoposide (10 µg/ml; prepared from stock in DMSO) for two hours. Nuclei were stained with DAPI (blue). Binary masks show the nuclear area used in subsequent analysis of the effect of etoposide on PLA. Yellow lines delineate nuclei as identified with a macro described in the Supplementary methods; each number indicates a region used to measure a single data point in the PLA channel. Fluorescence was detected with an Olympus Cell-R wide-field microscope, and scale bars represent 20 µm.

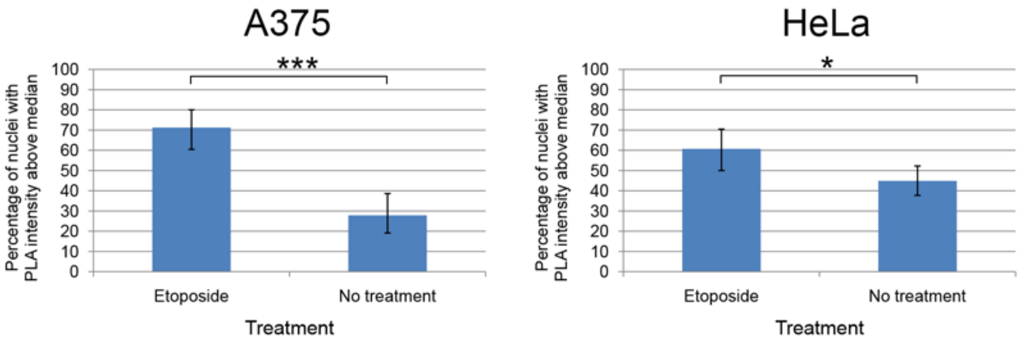

Supplementary figure 4: Effect of etoposide treatment on the p53•IL-1α interaction in A375 and HeLa cells, as detected by *in situ* proximity ligation assay (PLA). PLA was performed with mouse anti-IL-1α and rabbit anti-p53 antibodies either in A375 or HeLa cells treated with 10 µg/ml etoposide for two hours (n_{A375} = 80, n_{HeLa} = 84) or in control untreated cells (n_{A375} = 79, n_{HeLa} = 176). We determined the mean PLA fluorescence intensity over all pixels within the individual nuclei (I_n), counted the number of nuclei with I_n higher than median I_n within the each cell line-specific experiment and plotted the corresponding proportion of nuclei with higher I_n than median I_n in a given experiment. The statistical significance of the results was evaluated by Fisher's exact test (***, $p<0.001$; *, $p<0.05$); error bars represent 95% confidence intervals calculated by the adjusted Wald method. The PLA fluorescence was detected with a wide-field Olympus Cell-R microscope. To determine I_n of each nucleus, the images were processed in ImageJ with a macro described in the Supplementary methods. Primary data and all calculations are present in the Supplementary data file.

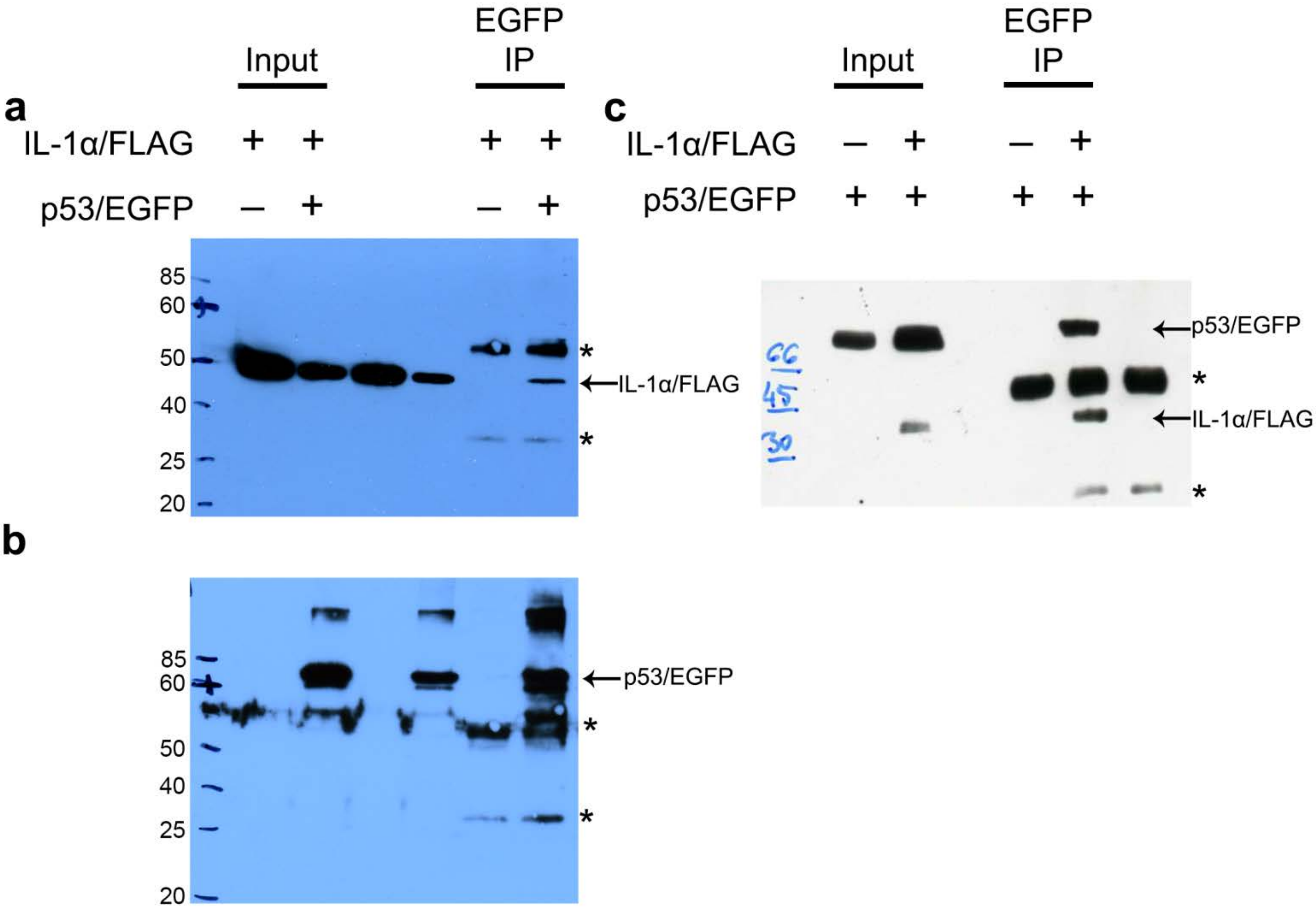

Supplementary figure 5: This figure shows full-size chemiluminescence records of the individual western blotting membranes, sections of which are depicted in the corresponding panels of Figure 2. Unlabeled lines belong either to biological replicates (panels a and b) or to negative controls (panel c). Asterisks represent heavy and light antibody chains. Numbers correspond to the MW of protein standards.

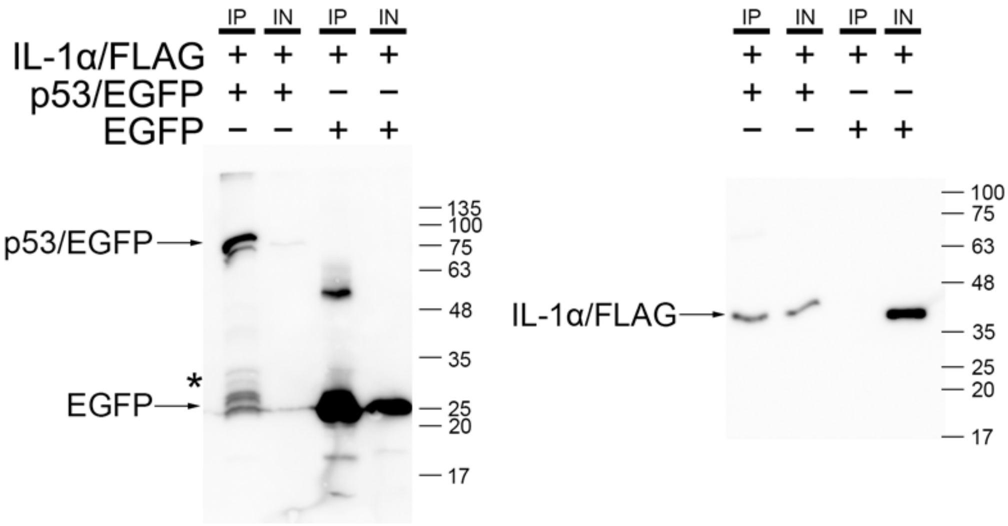

Supplementary figure 6: Coimmunoprecipitation of IL-1α and p53 in the U2OS cell line. Cells were transiently cotransfected with plasmids encoding Flag-tagged IL-1α (IL-1α/Flag) and either EGFP-tagged p53 (p53/EGFP) or EGFP alone. Coimmunoprecipitation was performed using a GFP-Trap agarose beads (ChromoTek) essentially according the manufacturer's recommendation. Amount of cells used for lysate preparation and subsequent input and IP load was the same as described for the other immunoprecipitation experiments with transfected cells. Separate membranes were developed with anti-EGFP and anti-Flag antibody respectively. Asterisk corresponds to possible p53/EGFP degradation products. Numbers represent protein MW standards. The experiment clearly confirmed association of IL-1α/Flag and p53/EGFP in the cells whereas EGFP alone did not precipitate any IL-1α/Flag from the transfected cells.

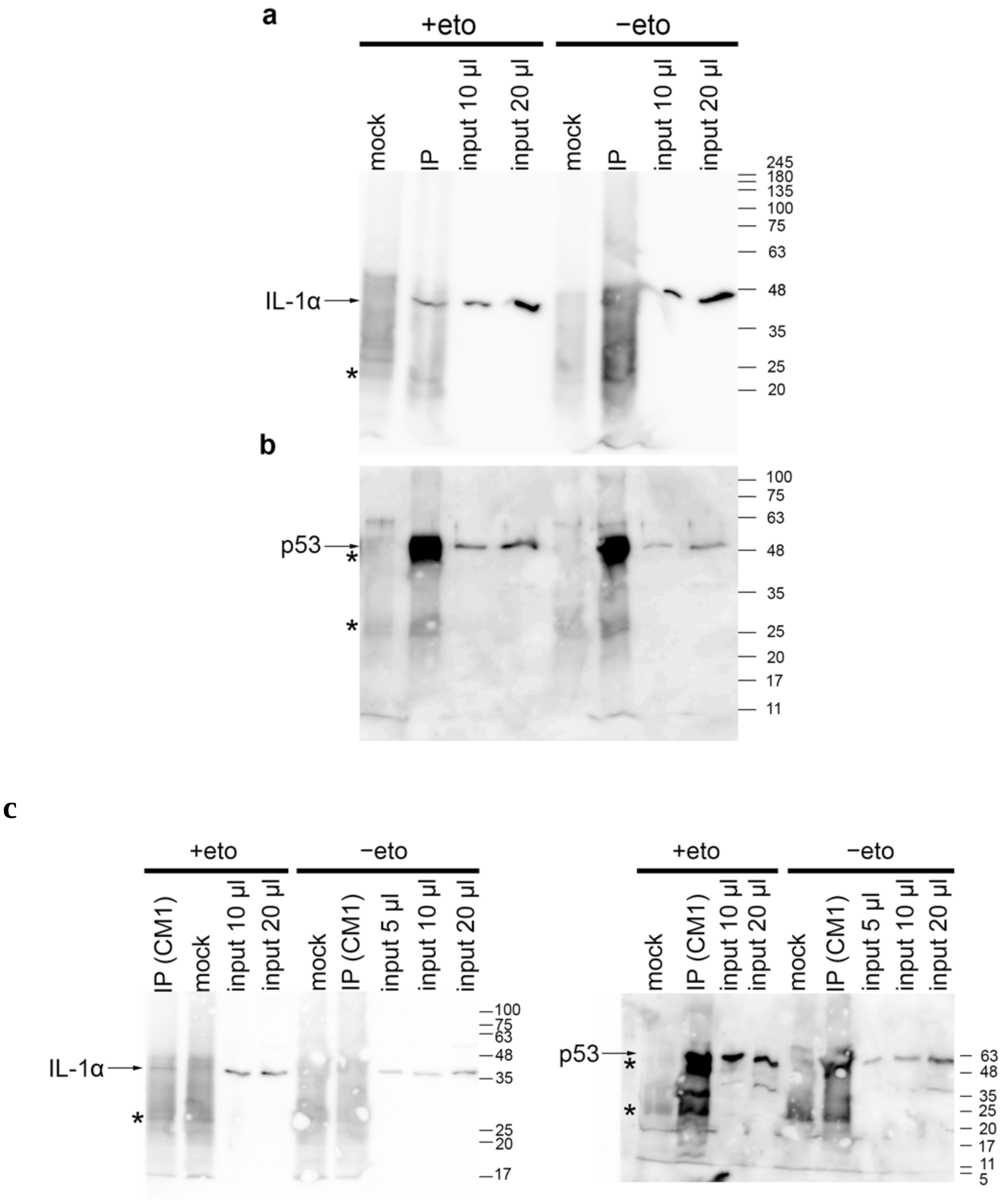

Supplementary figure 7: Endogenous IL-1α and p53 coimmunoprecipitate from the U2OS and A375 cell lysates upon etoposide treatment. Panels a) and b) are full-size versions of the records depicted in Figure 7 (U2OS cells). Panel c) shows coimmunoprecipitation experiments with A375 cells which were designed similarly to those in panels a) and b). Cells were either treated with etoposide (10 µg/ml, 2 hours, +eto) or left untreated (-eto). Co-immunoprecipitation (IP) was performed with anti-p53 antibody (CM-1) and resulting protein samples were split and analyzed for IL-1α and p53 content by western blotting in parallel. The IL-1α-specific bands are clearly visible in IPs from the etoposide-treated cells. Asterisks mark

heavy and light antibody chains. Numbers on the side correspond to the protein MW ladders. Mock IPs were performed from the same amount of the corresponding lysates without adding the CM-1 antibody.

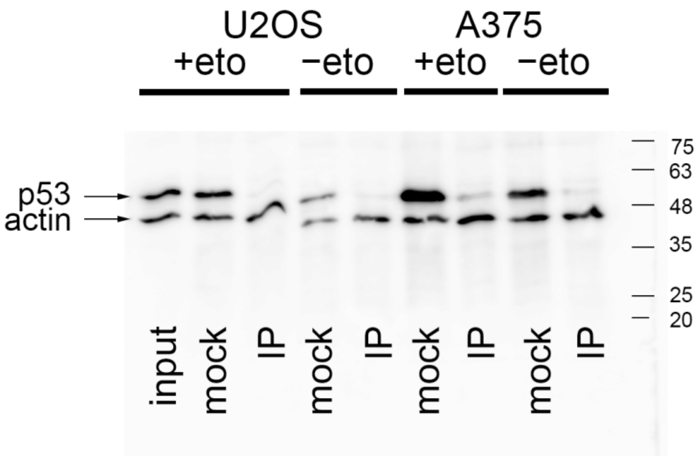

Supplementary figure 8: IP efficiency control. Equal aliquots of the supernatants from the IP experiments depicted in Figure 7 and Supplementary figure 7 were analyzed by western blotting. Membranes were stained simultaneously for β-actin and p53. The experiment documents high efficiency and specificity of the method. Most of the p53 protein was precipitated with the anti-53 CM-1 antibody whereas mock precipitation preserved p53 in the lysate. Input corresponds to the U2OS lysate from etoposide-treated cells.

Supplementary methods:

ImageJ macro used to detect position of nuclei (in blue channel) and to measure an average intensity in the red channel within the recognized region. The macro requires 8-bit RGB image.

```
currentTitle = getTitle(); //reads filename
run("Split Channels"); //splits RGB into 3 separate channels
selectWindow(currentTitle+" (blue)"); //select channel with DAPI
run("Gaussian Blur...", "sigma=3"); //blur to mask nuclear structures
setAutoThreshold("Huang dark"); //optimal automatic threshold for dark background
//run("Threshold...");
//setThreshold(37, 255);
setOption("BlackBackground", true);
run("Convert to Mask"); //turns blue channel into binary mask
run("Watershed"); //separate touching nuclei
run("Analyze Particles...", "size=1000-Infinity pixel circularity=0.60-1.00 add in_situ"); //exclude holes in nuclei, include nuclei touching edges of image, only sufficiently big and round nuclei are considered for analysis
selectWindow(currentTitle+" (red)");
roiManager("Measure"); //measures mean intensity for each nuclei
roiManager("Delete");
selectWindow(currentTitle+" (red)");
close();
selectWindow(currentTitle+" (blue)");
close();
selectWindow(currentTitle+" (green)");
close();
```

Supplementary data

Mean PLA intensities of individual nuclei as identified with macro described in the supplementary methods in three biological replicates. Median was calculated from the each biological replicate.

exp1-etoposide	exp1-no-treatment	exp1-merged	exp2-etoposide	exp2-no-treatment	exp2-merged	exp3-etoposide	exp3-no-treatment	exp3-merged
24.485	30.801	24.485	41.092	16.017	41.092	28.295	19.216	28.295
26.561	22.005	26.561	36.237	19.065	36.237	21.295	20.974	21.295
19.255	17.17	19.255	38.689	18.984	38.689	22.837	24.637	22.837
19.294	26.908	19.294	38.774	15.969	38.774	22.401	12.47	22.401
24.354	26.018	24.354	43.322	24.091	43.322	39.971	19.473	39.971
30.559	47.411	30.559	46.137	17.506	46.137	38.447	22.715	38.447
42.498	25.311	42.498	62.92	22.065	62.92	29.121	15.251	29.121
19.141	30.124	19.141	28.434	29.285	28.434	28.318	23.92	28.318
28.428	19.388	28.428	57.67	18.331	57.67	21.669	23.491	21.669
33.685	10.199	33.685	44.931	21.022	44.931	37.854	12.279	37.854
23.613	16.981	23.613	67.511	23.145	67.511	22.111	13.927	22.111
44.658	14.21	44.658	48.166	26.511	48.166	24.557	15.862	24.557
6.916	19.325	6.916	44.087	20.633	44.087	32.625	18.786	32.625
7.565	11.878	7.565	42.74	19.032	42.74	48.029	15.263	48.029
23.494	17.501	23.494	53.06	30.398	53.06	28.571	20.771	28.571
6.827	14.831	6.827	24.9	17.815	24.9	38.042	17.81	38.042
6.601	19.622	6.601	26.137	21.243	26.137	39.238	14.533	39.238
38.916	8.235	38.916	31.653	20.986	31.653	32.414	17.288	32.414
28.451	20.982	28.451	19.83	19.099	19.83	35.423	41.063	35.423
34.496	19.895	34.496	22.963	18.11	22.963	26.07	15.593	26.07
25.928	11.714	25.928	90.398	22.729	90.398	26.105	21.935	26.105
19.909	21.681	19.909	24.056	30.447	24.056	34.137	18.608	34.137
22.965	14.999	22.965	28.013	21.228	28.013	39.816	19.188	39.816
26.534	14.633	26.534	83.665	26.793	83.665	38.391	16.583	38.391
26.997	19.246	26.997	22.546	26.168	22.546	24.834	25.429	24.834
23.521	14.122	23.521	34.214	35.788	34.214	36.489	14.628	36.489
31.239	14.913	31.239	18.348	23.318	18.348	49.858	23.781	49.858
32.135	18.004	32.135	27.366	26.952	27.366	36.469	14.836	36.469
29.775	13.217	29.775	20.574	29.757	20.574	39.439	19.556	39.439
19.061	20.824	19.061	28.413	22.381	28.413	39.721	20.275	39.721
21.619	13.606	21.619	33.397	28.295	33.397	63.429	12.689	63.429
37.097	14.747	37.097	26.824	19.762	26.824	33.318	17.02	33.318
23.587	12.015	23.587	35.552	25.267	35.552	32.268	16.545	32.268
50.495	13.667	50.495	31.696	17.331	31.696	28.823	16.695	28.823
17.506	13.098	17.506	27.49	19.375	27.49	19.427	18.688	19.427
13.609	17.639	13.609	28.97	18.293	28.97	17.331	26.559	17.331
46.108	10.272	46.108	36.065	20.812	36.065	29.228	20.591	29.228
35.762	10.998	35.762	48.908	18.101	48.908	20.552	19.141	20.552
21.191	16.033	21.191	25.887	32.744	25.887	39.165	25.769	39.165
25.763	14.322	25.763	35.128	28.341	35.128	28.672	19.833	28.672
26.297	23.072	26.297	24.035	21.014	24.035	24.55	18.406	24.55
48.544	7.719	48.544	34.537	32.29	34.537	18.587	20.209	18.587
38.323	7.102	38.323	33.691	35.748	33.691	24.957	23.816	24.957
29.472	16.449	29.472	35.543	24.857	35.543	29.635	24.474	29.635
19.811	14.141	19.811	28.169	18.698	28.169	4.619	16.333	4.619

22.061	16.291	22.061	12.767	15.409	12.767	15.387	20.072	15.387
22.844	9.679	22.844	32.389	18.202	32.389	12.395	21.849	12.395
27.397	12.631	27.397	32.575	13.855	32.575	13.901	20.579	13.901
24.88	10.856	24.88	24.882	19.305	24.882	13.763	20.326	13.763
24.091	14.377	24.091	37.018	21.933	37.018	12.783	21.667	12.783
19.681	9.377	19.681	27.959	18.043	27.959	12.654	16.343	12.654
19.83	9.882	19.83	38.049	17.749	38.049	13.765	16.123	13.765
31.004	13.917	31.004	24.13	25.349	24.13	17.802	17.305	17.802
27.097	20.314	27.097	39.173	23.125	39.173	11.334	18.317	11.334
25.901	22.937	25.901	35.449	20.223	35.449	18.792	24.391	18.792
36.464	22.903	36.464	38.978	18.341	38.978	16.581	17.246	16.581
27.549	14.804	27.549	35.51	16.769	35.51	13.549	13.933	13.549
30.17	11.076	30.17	49.535	24.856	49.535	9.716	25.594	9.716
20.792	26.557	20.792	28.461		28.461	12.692	15.899	12.692
36.541	17.656	36.541	26.918		26.918	12.722	15.983	12.722
29.442	11.902	29.442	38.762		38.762	21.746	25.67	21.746
14.203	14.921	14.203	49.688		49.688	13.952	14.681	13.952
36.794	17.524	36.794	26.742		26.742	13.205	21.311	13.205
25.73	17.629	25.73			16.017	14.532	17.467	14.532
35.092	16.723	35.092			19.065	22.309	24.078	22.309
17.606	17.209	17.606			18.984	33.002	26.651	33.002
27.148	12.958	27.148			15.969	24.037	25.083	24.037
17.895	21.961	17.895			24.091	20.171	29.636	20.171
17.703	14.891	17.703			17.506	33.461	27.377	33.461
30.126	13.77	30.126			22.065	28.4	24.108	28.4
21.755	14.508	21.755			29.285	37.773	27.975	37.773
32.1	26.735	32.1			18.331	24.347	21.106	24.347
25.255	59.247	25.255			21.022	35.063	28.934	35.063
27.941	63.062	27.941			23.145	38.098	27.355	38.098
21.014	69.713	21.014			26.511	24.421	17.473	24.421
37.274	18.528	37.274			20.633	33.109	13.699	33.109
17.628	15.827	17.628			19.032	19.65	21.356	19.65
32.162	12.449	32.162			30.398	17.309	21.41	17.309
30.477	10.268	30.477			17.815	29.925	13.748	29.925
16.863	13.878	16.863			21.243	19.505	12.511	19.505
22.57	11.84	22.57			20.986	47.217	13.727	47.217
14.053	10.588	14.053			19.099	53.248	20.983	53.248
111.25	12.196	111.25			18.11	39.923	21.777	39.923
32.832	9.959	32.832			22.729	33.633	16.773	33.633
97.811	11.093	97.811			30.447	43.231	17.958	43.231
23.802	18.08	23.802			21.228	35.496	19.817	35.496
31.433	15.831	31.433			26.793	51.267	19.914	51.267
21.628	14.83	21.628			26.168	43.448	17.049	43.448
20.282	16.593	20.282			35.788	34.233	13.279	34.233
53.571	16.666	53.571			23.318	28.71	13.619	28.71
28.424	19.851	28.424			26.952	31.897	13.279	31.897
26.799	13.434	26.799			29.757	31.029	24.107	31.029
32.152	16.211	32.152			22.381	23.872	23.545	23.872
27.512	21.542	27.512			28.295	24.29	21.522	24.29
34.133	20.667	34.133			19.762	35.86	19.099	35.86

32.354	10.551	32.354		25.267	36.712	19.44	36.712
45.119	18.724	45.119		17.331	33.348	14.995	33.348
18.676	16.646	18.676		19.375	27.256	17.756	27.256
23.75	13.813	23.75		18.293	29.667	21.136	29.667
35.71	10.269	35.71		20.812	42.804	20.741	42.804
37.076	15.283	37.076		18.101	48.586	15.7	48.586
48.392	15.763	48.392		32.744	41.548	22.889	41.548
24.973	12.617	24.973		28.341	42.367	38.898	42.367
19.631	14.587	19.631		21.014	56.099	21.755	56.099
17.124	18.18	17.124		32.29	42.242	21.566	42.242
21.853	13.105	21.853		35.748	38.593	21.995	38.593
51.819	7.038	51.819		24.857	34.374	21.093	34.374
20.442	11.148	20.442		18.698	25.581	20.685	25.581
17.613	13.167	17.613		15.409	31.794	36.328	31.794
18.398	15.043	18.398		18.202	43.777	18.437	43.777
27.833	19.638	27.833		13.855	15.614	23.02	15.614
32.044	11.372	32.044		19.305	29.851	22.107	29.851
27.427	14.8	27.427		21.933	32.473	23.59	32.473
22.908	17.37	22.908		18.043	38.594	32.398	38.594
31.822	15.14	31.822		17.749	40.301	16.803	40.301
83.043	12.48	83.043		25.349	35.918	20.297	35.918
20.04	10.834	20.04		23.125	34.298	23.864	34.298
58.475		58.475		20.223	39.972	24.119	39.972
15.995		15.995		18.341	32.854	19.935	32.854
48.365		48.365		16.769	37.8	22.157	37.8
22.855		22.855		24.856	37.365	23.767	37.365
74.997		74.997	median	26.793	38.217	9.946	38.217
24.039		24.039			40.083	17.393	40.083
19.113		19.113			49.326	15.603	49.326
25.525		25.525			26.784	16.365	26.784
19.882		19.882			34.377	17.086	34.377
32.879		32.879			36.457	22.367	36.457
33.568		33.568			56.956	14.371	56.956
25.942		25.942			43.583	16.565	43.583
20.024		20.024			42.69	15.325	42.69
30.799		30.799			31.483	14.612	31.483
22.74		22.74			38.153	18.393	38.153
19.983		19.983			35.255	17.247	35.255
16.271		16.271			27.812	12.478	27.812
20.584		20.584			29.981	16.467	29.981
17.415		17.415			43.367	15.419	43.367
22.65		22.65			46.862	17.59	46.862
24.536		24.536			35.903	11.272	35.903
39.776		39.776				14.75	19.216
25.87		25.87				11.102	20.974
31.608		31.608				12.563	24.637
27.634		27.634				13.838	12.47
35.199		35.199				14.266	19.473
42.095		42.095				16.975	22.715
35.88		35.88				20.682	15.251

24.886	24.886	22.896	23.92
26.366	26.366		23.491
46.388	46.388		12.279
18.649	18.649		13.927
22.279	22.279		15.862
13.603	13.603		18.786
27.48	27.48		15.263
24.789	24.789		20.771
24.568	24.568		17.81
24	24		14.533
38.178	38.178		17.288
33.8	33.8		41.063
27.849	27.849		15.593
29.282	29.282		21.935
28.932	28.932		18.608
30.031	30.031		19.188
20.852	20.852		16.583
25.261	25.261		25.429
	30.801		14.628
	22.005		23.781
	17.17		14.836
	26.908		19.556
	26.018		20.275
	47.411		12.689
	25.311		17.02
	30.124		16.545
	19.388		16.695
	10.199		18.688
	16.981		26.559
	14.21		20.591
	19.325		19.141
	11.878		25.769
	17.501		19.833
	14.831		18.406
	19.622		20.209
	8.235		23.816
	20.982		24.474
	19.895		16.333
	11.714		20.072
	21.681		21.849
	14.999		20.579
	14.633		20.326
	19.246		21.667
	14.122		16.343
	14.913		16.123
	18.004		17.305
	13.217		18.317
	20.824		24.391
	13.606		17.246
	14.747		13.933

12.015	25.594
13.667	15.899
13.098	15.983
17.639	25.67
10.272	14.681
10.998	21.311
16.033	17.467
14.322	24.078
23.072	26.651
7.719	25.083
7.102	29.636
16.449	27.377
14.141	24.108
16.291	27.975
9.679	21.106
12.631	28.934
10.856	27.355
14.377	17.473
9.377	13.699
9.882	21.356
13.917	21.41
20.314	13.748
22.937	12.511
22.903	13.727
14.804	20.983
11.076	21.777
26.557	16.773
17.656	17.958
11.902	19.817
14.921	19.914
17.524	17.049
17.629	13.279
16.723	13.619
17.209	13.279
12.958	24.107
21.961	23.545
14.891	21.522
13.77	19.099
14.508	19.44
26.735	14.995
59.247	17.756
63.062	21.136
69.713	20.741
18.528	15.7
15.827	22.889
12.449	38.898
10.268	21.755
13.878	21.566
11.84	21.995
10.588	21.093

	12.196		20.685
	9.959		36.328
	11.093		18.437
	18.08		23.02
	15.831		22.107
	14.83		23.59
	16.593		32.398
	16.666		16.803
	19.851		20.297
	13.434		23.864
	16.211		24.119
	21.542		19.935
	20.667		22.157
	10.551		23.767
	18.724		9.946
	16.646		17.393
	13.813		15.603
	10.269		16.365
	15.283		17.086
	15.763		22.367
	12.617		14.371
	14.587		16.565
	18.18		15.325
	13.105		14.612
	7.038		18.393
	11.148		17.247
	13.167		12.478
	15.043		16.467
	19.638		15.419
	11.372		17.59
	14.8		11.272
	17.37		14.75
	15.14		11.102
	12.48		12.563
	10.834		13.838
median	20.998		14.266
			16.975
			20.682
			22.896
		median	22.233

Mean PLA intensities of individual nuclei normalized to median calculated for the each biological replicate. Data from etoposide treated and nontreated cells were merged together for further analysis in the last two collums.

exp1-eto	exp1-no-tr	exp1-merged	exp2-eto	exp2-no-tr	exp2-merged	exp3-eto	exp3-no	exp3-merged	Eto-median-merge	no-tr-median-merge
1.166	1.467	1.166	1.534	0.598	1.534	1.273	0.864	1.273	1.166063435	1.466853986
1.265	1.048	1.265	1.352	0.712	1.352	0.958	0.943	0.958	1.264929993	1.047956948
0.917	0.818	0.917	1.444	0.709	1.444	1.027	1.108	1.027	0.916992094	0.817696924
0.919	1.281	0.919	1.447	0.596	1.447	1.008	0.561	1.008	0.918849414	1.281455377
1.160	1.239	1.160	1.617	0.899	1.617	1.798	0.876	1.798	1.159824745	1.239070388
1.455	2.258	1.455	1.722	0.653	1.722	1.729	1.022	1.729	1.455329079	2.257881703
2.024	1.205	2.024	2.348	0.824	2.348	1.310	0.686	1.310	2.023907039	1.205400514
0.912	1.435	0.912	1.061	1.093	1.061	1.274	1.076	1.274	0.911563006	1.43461282
1.354	0.923	1.354	2.152	0.684	2.152	0.975	1.057	0.975	1.353843223	0.923326031
1.604	0.486	1.604	1.677	0.785	1.677	1.703	0.552	1.703	1.6042004	0.485712925
1.125	0.809	1.125	2.520	0.864	2.520	0.995	0.626	0.995	1.12453567	0.808696066
2.127	0.677	2.127	1.798	0.989	1.798	1.105	0.713	1.105	2.126773978	0.676731117
0.329	0.920	0.329	1.645	0.770	1.645	1.467	0.845	1.467	0.329364701	0.920325745
0.360	0.566	0.360	1.595	0.710	1.595	2.160	0.687	2.160	0.360272407	0.565672921
1.119	0.833	1.119	1.980	1.135	1.980	1.285	0.934	1.285	1.118868464	0.83346033
0.325	0.706	0.325	0.929	0.665	0.929	1.711	0.801	1.711	0.325126202	0.706305362
0.314	0.934	0.314	0.976	0.793	0.976	1.765	0.654	1.765	0.314363273	0.93446995
1.853	0.392	1.853	1.181	0.783	1.181	1.458	0.778	1.458	1.853319364	0.392180208
1.355	0.999	1.355	0.740	0.713	0.740	1.593	1.847	1.593	1.354938566	0.999238023
1.643	0.947	1.643	0.857	0.676	0.857	1.173	0.701	1.173	1.642823126	0.947471188
1.235	0.558	1.235	3.374	0.848	3.374	1.174	0.987	1.174	1.234784265	0.557862654
0.948	1.033	0.948	0.898	1.136	0.898	1.535	0.837	1.535	0.948137918	1.032526907
1.094	0.714	1.094	1.046	0.792	1.046	1.791	0.863	1.791	1.093675588	0.714306124
1.264	0.697	1.264	3.123	1.000	3.123	1.727	0.746	1.727	1.263644157	0.696875893
1.286	0.917	1.286	0.841	0.977	0.841	1.117	1.144	1.117	1.285693876	0.916563482
1.120	0.673	1.120	1.277	1.336	1.277	1.641	0.658	1.641	1.1201543	0.672540242
1.488	0.710	1.488	0.685	0.870	0.685	2.243	1.070	2.243	1.487713116	0.710210496
1.530	0.857	1.530	1.021	1.006	1.021	1.640	0.667	1.640	1.530383846	0.857414992
1.418	0.629	1.418	0.768	1.111	0.768	1.774	0.880	1.774	1.41799219	0.629440899
0.908	0.992	0.908	1.060	0.835	1.060	1.787	0.912	1.787	0.907753119	0.991713497
1.030	0.648	1.030	1.246	1.056	1.246	2.853	0.571	2.853	1.029574245	0.647966473
1.767	0.702	1.767	1.001	0.738	1.001	1.499	0.766	1.499	1.766692066	0.702304981
1.123	0.572	1.123	1.327	0.943	1.327	1.451	0.744	1.451	1.123297457	0.572197352
2.405	0.651	2.405	1.183	0.647	1.183	1.296	0.751	1.296	2.404752834	0.650871512
0.834	0.624	0.834	1.026	0.723	1.026	0.874	0.841	0.874	0.833698447	0.623773693
0.648	0.840	0.648	1.081	0.683	1.081	0.780	1.195	0.780	0.648109344	0.840032384
2.196	0.489	2.196	1.346	0.777	1.346	1.315	0.926	1.315	2.195828174	0.489189447
1.703	0.524	1.703	1.825	0.676	1.825	0.924	0.861	0.924	1.703114582	0.523764168
1.009	0.764	1.009	0.966	1.222	0.966	1.762	1.159	1.762	1.009191352	0.763548909
1.227	0.682	1.227	1.311	1.058	1.311	1.290	0.892	1.290	1.226926374	0.682064959
1.252	1.099	1.252	0.897	0.784	0.897	1.104	0.828	1.104	1.252357367	1.098771312
2.312	0.368	2.312	1.289	1.205	1.289	0.836	0.909	0.836	2.311839223	0.367606439
1.825	0.338	1.825	1.257	1.334	1.257	1.123	1.071	1.123	1.825078579	0.338222688
1.404	0.783	1.404	1.327	0.928	1.327	1.333	1.101	1.333	1.403562244	0.78336032

0.943	0.673	0.943	1.051	0.698	1.051	0.208	0.735	0.208	0.943470807	0.67344509
1.051	0.776	1.051	0.477	0.575	0.477	0.692	0.903	0.692	1.050623869	0.775835794
1.088	0.461	1.088	1.209	0.679	1.209	0.558	0.983	0.558	1.087913135	0.460948662
1.305	0.602	1.305	1.216	0.517	1.216	0.625	0.926	0.625	1.304743309	0.601533479
1.185	0.517	1.185	0.929	0.721	0.929	0.619	0.914	0.619	1.18487475	0.517001619
1.147	0.685	1.147	1.382	0.819	1.382	0.575	0.975	0.575	1.147299743	0.684684256
0.937	0.447	0.937	1.044	0.673	1.044	0.569	0.735	0.569	0.937279741	0.44656634
0.944	0.471	0.944	1.420	0.662	1.420	0.619	0.725	0.619	0.944375655	0.470616249
1.477	0.663	1.477	0.901	0.946	0.901	0.801	0.778	0.801	1.476521573	0.662777407
1.290	0.967	1.290	1.462	0.863	1.462	0.510	0.824	0.510	1.290456234	0.967425469
1.233	1.092	1.233	1.323	0.755	1.323	0.845	1.097	0.845	1.233498428	1.092342128
1.737	1.091	1.737	1.455	0.685	1.455	0.746	0.776	0.746	1.736546338	1.090722926
1.312	0.705	1.312	1.325	0.626	1.325	0.609	0.627	0.609	1.311982094	0.705019526
1.437	0.527	1.437	1.849	0.928	1.849	0.437	1.151	0.437	1.436803505	0.527478808
0.990	1.265	0.990	1.062	0.000	1.062	0.571	0.715	0.571	0.990189542	1.264739499
1.740	0.841	1.740	1.005	0.000	1.005	0.572	0.719	0.572	1.740213354	0.840841985
1.402	0.567	1.402	1.447	0.000	1.447	0.978	1.155	0.978	1.402133537	0.566815887
0.676	0.711	0.676	1.855	0.000	1.855	0.628	0.660	0.628	0.676397752	0.710591485
1.752	0.835	1.752	0.998	0.000	0.998	0.594	0.959	0.594	1.75226212	0.834555672
1.225	0.840	1.225	0.000	0.000	0.598	0.654	0.786	0.654	1.225354796	0.839556148
1.671	0.796	1.671	0.000	0.000	0.712	1.003	1.083	1.003	1.671206782	0.796409182
0.838	0.820	0.838	0.000	0.000	0.709	1.484	1.199	1.484	0.838460806	0.819554243
1.293	0.617	1.293	0.000	0.000	0.596	1.081	1.128	1.081	1.292885037	0.617106391
0.852	1.046	0.852	0.000	0.000	0.899	0.907	1.333	0.907	0.852224021	1.045861511
0.843	0.709	0.843	0.000	0.000	0.653	1.505	1.231	1.505	0.843080293	0.709162777
1.435	0.656	1.435	0.000	0.000	0.824	1.277	1.084	1.277	1.434708067	0.655776741
1.036	0.691	1.036	0.000	0.000	1.093	1.699	1.258	1.699	1.036051052	0.690922945
1.529	1.273	1.529	0.000	0.000	0.684	1.095	0.949	1.095	1.528717021	1.273216497
1.203	2.822	1.203	0.000	0.000	0.785	1.577	1.301	1.577	1.202733594	2.821554434
1.331	3.003	1.331	0.000	0.000	0.864	1.714	1.230	1.714	1.330650538	3.003238404
1.001	3.320	1.001	0.000	0.000	0.989	1.098	0.786	1.098	1.000761977	3.319982856
1.775	0.882	1.775	0.000	0.000	0.770	1.489	0.616	1.489	1.77512144	0.882369749
0.840	0.754	0.840	0.000	0.000	0.710	0.884	0.961	0.884	0.839508525	0.753738451
1.532	0.593	1.532	0.000	0.000	1.135	0.779	0.963	0.779	1.531669683	0.592865987
1.451	0.489	1.451	0.000	0.000	0.665	1.346	0.618	1.346	1.451423945	0.488998952
0.803	0.661	0.803	0.000	0.000	0.793	0.877	0.563	0.877	0.803076483	0.660920088
1.075	0.564	1.075	0.000	0.000	0.783	2.124	0.617	2.124	1.074864273	0.563863225
0.669	0.504	0.669	0.000	0.000	0.713	2.395	0.944	2.395	0.669254215	0.504238499
5.298	0.581	5.298	0.000	0.000	0.676	1.796	0.979	1.796	5.298123631	0.580817221
1.564	0.474	1.564	0.000	0.000	0.848	1.513	0.754	1.513	1.563577484	0.474283265
4.658	0.528	4.658	0.000	0.000	1.136	1.944	0.808	1.944	4.658110296	0.528288408
1.134	0.861	1.134	0.000	0.000	0.792	1.597	0.891	1.597	1.133536527	0.861034384
1.497	0.754	1.497	0.000	0.000	1.000	2.306	0.896	2.306	1.496952091	0.753928946
1.030	0.706	1.030	0.000	0.000	0.977	1.954	0.767	1.954	1.030002857	0.706257739
0.966	0.790	0.966	0.000	0.000	1.336	1.540	0.597	1.540	0.965901514	0.790218116
2.551	0.794	2.551	0.000	0.000	0.870	1.291	0.613	1.291	2.551242976	0.793694638
1.354	0.945	1.354	0.000	0.000	1.006	1.435	0.597	1.435	1.353652729	0.94537575
1.276	0.640	1.276	0.000	0.000	1.111	1.396	1.084	1.396	1.276264406	0.639775217
1.531	0.772	1.531	0.000	0.000	0.835	1.074	1.059	1.074	1.531193447	0.772025907
1.310	1.026	1.310	0.000	0.000	1.056	1.093	0.968	1.093	1.310220021	1.025907229

1.626	0.984	1.626	0.000	0.000	0.738	1.613	0.859	1.613	1.625535765	0.984236594
1.541	0.502	1.541	0.000	0.000	0.943	1.651	0.874	1.651	1.540813411	0.502476426
2.149	0.892	2.149	0.000	0.000	0.647	1.500	0.674	1.500	2.14872845	0.891703972
0.889	0.793	0.889	0.000	0.000	0.723	1.226	0.799	1.226	0.88941804	0.792742166
1.131	0.658	1.131	0.000	0.000	0.683	1.334	0.951	1.334	1.131060101	0.657824555
1.701	0.489	1.701	0.000	0.000	0.777	1.925	0.933	1.925	1.700638156	0.489046576
1.766	0.728	1.766	0.000	0.000	0.676	2.185	0.706	2.185	1.765691971	0.727831222
2.305	0.751	2.305	0.000	0.000	1.222	1.869	1.030	1.869	2.304600438	0.750690542
1.189	0.601	1.189	0.000	0.000	1.058	1.906	1.750	1.906	1.189303743	0.600866749
0.935	0.695	0.935	0.000	0.000	0.784	2.523	0.979	2.523	0.934898562	0.694685208
0.816	0.866	0.816	0.000	0.000	1.205	1.900	0.970	1.900	0.815506239	0.865796743
1.041	0.624	1.041	0.000	0.000	1.334	1.736	0.989	1.736	1.040718164	0.624107058
2.468	0.335	2.468	0.000	0.000	0.928	1.546	0.949	1.546	2.467806458	0.335174779
0.974	0.531	0.974	0.000	0.000	0.698	1.151	0.930	1.151	0.973521288	0.530907705
0.839	0.627	0.839	0.000	0.000	0.575	1.430	1.634	1.430	0.838794171	0.62705972
0.876	0.716	0.876	0.000	0.000	0.679	1.969	0.829	1.969	0.876178684	0.716401562
1.326	0.935	1.326	0.000	0.000	0.517	0.702	1.035	0.702	1.325507191	0.935231927
1.526	0.542	1.526	0.000	0.000	0.721	1.343	0.994	1.343	1.5260501	0.541575388
1.306	0.705	1.306	0.000	0.000	0.819	1.461	1.061	1.461	1.306172016	0.704829031
1.091	0.827	1.091	0.000	0.000	0.673	1.736	1.457	1.736	1.090961044	0.82722164
1.515	0.721	1.515	0.000	0.000	0.662	1.813	0.756	1.813	1.515477665	0.72102105
3.955	0.594	3.955	0.000	0.000	0.946	1.616	0.913	1.616	3.95480522	0.594342318
0.954	0.516	0.954	0.000	0.000	0.863	1.543	1.073	1.543	0.954376607	0.5159539
2.785	0.000	2.785	0.000	0.000	0.755	1.798	1.085	1.798	2.784789028	0.597805397
0.762	0.000	0.762	0.000	0.000	0.685	1.478	0.897	1.478	0.761739213	0.711566454
2.303	0.000	2.303	0.000	0.000	0.626	1.700	0.997	1.700	2.303314601	0.708543276
1.088	0.000	1.088	0.000	0.000	0.928	1.681	1.069	1.681	1.088436994	0.596013884
3.572	0.000	3.572		median	1.000	1.719	0.447	1.719	3.571625869	0.899152764
1.145	0.000	1.145				1.803	0.782	1.803	1.144823317	0.653379614
0.910	0.000	0.910				2.219	0.702	2.219	0.910229546	0.823535998
1.216	0.000	1.216				1.205	0.736	1.205	1.215591961	1.093009368
0.947	0.000	0.947				1.546	0.768	1.546	0.946852081	0.684171239
1.566	0.000	1.566				1.640	1.006	1.640	1.565815792	0.78460792
1.599	0.000	1.599				2.562	0.646	2.562	1.598628441	0.863845034
1.235	0.000	1.235				1.960	0.745	1.960	1.235450995	0.989474863
0.954	0.000	0.954				1.920	0.689	1.920	0.95361463	0.770089202
1.467	0.000	1.467				1.416	0.657	1.416	1.466758739	0.710334789
1.083	0.000	1.083				1.716	0.827	1.716	1.082960282	1.134550069
0.952	0.000	0.952				1.586	0.776	1.586	0.951662063	0.664912477
0.775	0.000	0.775				1.251	0.561	1.251	0.774883322	0.792856343
0.980	0.000	0.980				1.348	0.741	1.348	0.980283837	0.783264285
0.829	0.000	0.829				1.951	0.694	1.951	0.829364701	0.712835442
1.079	0.000	1.079				2.108	0.791	2.108	1.078674159	0.675922816
1.168	0.000	1.168				1.615	0.507	1.615	1.168492237	0.848318591
1.894	0.000	1.894				0.000	0.663	0.864	1.894275645	1.136378905
1.232	0.000	1.232				0.000	0.499	0.943	1.232022097	0.792296495
1.505	0.000	1.505				0.000	0.565	1.108	1.505286218	1
1.316	0.000	1.316				0.000	0.622	0.561	1.316030098	0.976673012
1.676	0.000	1.676				0.000	0.642	0.876	1.676302505	1.335722017
2.005	0.000	2.005				0.000	0.764	1.022	2.004714735	0.870301945

1.709	0.000	1.709	0.000	0.930	0.686	1.708734165	1.005934386
1.185	0.000	1.185	0.000	1.030	1.076	1.185160491	1.11062591
1.256	0.000	1.256	0.000	0.000	1.057	1.255643395	0.835330124
2.209	0.000	2.209	0.000	0.000	0.552	2.209162777	1.056059419
0.888	0.000	0.888	0.000	0.000	0.626	0.888132203	0.737580711
1.061	0.000	1.061	0.000	0.000	0.713	1.06100581	0.943044825
0.648	0.000	0.648	0.000	0.000	0.845	0.647823602	0.646848057
1.309	0.000	1.309	0.000	0.000	0.687	1.308696066	0.72313664
1.181	0.000	1.181	0.000	0.000	0.934	1.180541004	0.682752958
1.170	0.000	1.170	0.000	0.000	0.801	1.170016192	0.776770052
1.143	0.000	1.143	0.000	0.000	0.654	1.142965997	0.675586907
1.818	0.000	1.818	0.000	0.000	0.778	1.818173159	1.222110253
1.610	0.000	1.610	0.000	0.000	1.847	1.609677112	1.057776285
1.326	0.000	1.326	0.000	0.000	0.701	1.326269168	0.784309335
1.395	0.000	1.395	0.000	0.000	0.987	1.394513763	1.205165528
1.378	0.000	1.378	0.000	0.000	0.837	1.377845509	1.33422909
1.430	0.000	1.430	0.000	0.000	0.863	1.430183827	0.927742321
0.993	0.000	0.993	0.000	0.000	0.746	0.993046957	0.697868846
1.203	0.000	1.203	0.000	0.000	1.144	1.203019335	0.575112903
0.000	0.000	1.467	0.000	0.000	0.658	1.533684171	0.679356548
0.000	0.000	1.048	0.000	0.000	1.070	1.352480125	0.517112679
0.000	0.000	0.818	0.000	0.000	0.667	1.443996566	0.720524017
0.000	0.000	1.281	0.000	0.000	0.880	1.447169037	0.818609338
0.000	0.000	1.239	0.000	0.000	0.912	1.616914866	0.673422163
0.000	0.000	2.258	0.000	0.000	0.571	1.721979622	0.662449147
0.000	0.000	1.205	0.000	0.000	0.766	2.348374575	0.946105326
0.000	0.000	1.435	0.000	0.000	0.744	1.061247341	0.863098571
0.000	0.000	0.923	0.000	0.000	0.751	2.152427873	0.754786698
0.000	0.000	0.486	0.000	0.000	0.841	1.676967865	0.684544471
0.000	0.000	0.809	0.000	0.000	1.195	2.519725301	0.625872429
0.000	0.000	0.677	0.000	0.000	0.926	1.797708357	0.927704998
0.000	0.000	0.920	0.000	0.000	0.861	1.6454671	0.864300814
0.000	0.000	0.566	0.000	0.000	1.159	1.595192774	0.943372464
0.000	0.000	0.833	0.000	0.000	0.892	1.980368007	1.108127558
0.000	0.000	0.706	0.000	0.000	0.828	0.929347218	0.560877974
0.000	0.000	0.934	0.000	0.000	0.909	0.975515993	0.875860208
0.000	0.000	0.392	0.000	0.000	1.071	1.181390662	1.021679485
0.000	0.000	0.999	0.000	0.000	1.101	0.740118688	0.685962308
0.000	0.000	0.947	0.000	0.000	0.735	0.857052215	1.075878199
0.000	0.000	0.558	0.000	0.000	0.903	3.373940955	1.056582557
0.000	0.000	1.033	0.000	0.000	0.983	0.897846452	0.552287141
0.000	0.000	0.714	0.000	0.000	0.926	1.045534281	0.626411191
0.000	0.000	0.697	0.000	0.000	0.914	3.122643974	0.71344398
0.000	0.000	0.917	0.000	0.000	0.975	0.841488448	0.844960194
0.000	0.000	0.673	0.000	0.000	0.735	1.276975329	0.686502047
0.000	0.000	0.710	0.000	0.000	0.725	0.684805733	0.934241893
0.000	0.000	0.857	0.000	0.000	0.778	1.021386183	0.801061485
0.000	0.000	0.629	0.000	0.000	0.824	0.767887135	0.653667971
0.000	0.000	0.992	0.000	0.000	1.097	1.060463554	0.777582872
0.000	0.000	0.648	0.000	0.000	0.776	1.24648229	1.846939234

0.000	0.000	0.702
0.000	0.000	0.572
0.000	0.000	0.651
0.000	0.000	0.624
0.000	0.000	0.840
0.000	0.000	0.489
0.000	0.000	0.524
0.000	0.000	0.764
0.000	0.000	0.682
0.000	0.000	1.099
0.000	0.000	0.368
0.000	0.000	0.338
0.000	0.000	0.783
0.000	0.000	0.673
0.000	0.000	0.776
0.000	0.000	0.461
0.000	0.000	0.602
0.000	0.000	0.517
0.000	0.000	0.685
0.000	0.000	0.447
0.000	0.000	0.471
0.000	0.000	0.663
0.000	0.000	0.967
0.000	0.000	1.092
0.000	0.000	1.091
0.000	0.000	0.705
0.000	0.000	0.527
0.000	0.000	1.265
0.000	0.000	0.841
0.000	0.000	0.567
0.000	0.000	0.711
0.000	0.000	0.835
0.000	0.000	0.840
0.000	0.000	0.796
0.000	0.000	0.820
0.000	0.000	0.617
0.000	0.000	1.046
0.000	0.000	0.709
0.000	0.000	0.656
0.000	0.000	0.691
0.000	0.000	1.273
0.000	0.000	2.822
0.000	0.000	3.003
0.000	0.000	3.320
0.000	0.000	0.882
0.000	0.000	0.754
0.000	0.000	0.593
0.000	0.000	0.489
0.000	0.000	0.661
0.000	0.000	0.564

0.000	0.000	0.627	1.001157019	0.701344848
0.000	0.000	1.151	1.326913746	0.986596501
0.000	0.000	0.715	1.182995559	0.836954077
0.000	0.000	0.719	1.026014257	0.863041425
0.000	0.000	1.155	1.081252566	0.745873251
0.000	0.000	0.660	1.346060538	1.143750281
0.000	0.000	0.959	1.825402157	0.657940899
0.000	0.000	0.786	0.966185198	1.069626231
0.000	0.000	1.083	1.311088717	0.667296361
0.000	0.000	1.199	0.897062666	0.879593397
0.000	0.000	1.128	1.289030717	0.911932713
0.000	0.000	1.333	1.257455305	0.570728197
0.000	0.000	1.231	1.326577837	0.765528719
0.000	0.000	1.084	1.051356698	0.74416408
0.000	0.000	1.258	0.476505057	0.750910808
0.000	0.000	0.949	1.208860523	0.840552332
0.000	0.000	1.301	1.215802635	1.194575631
0.000	0.000	1.230	0.9286754	0.926145819
0.000	0.000	0.786	1.38162953	0.86092745
0.000	0.000	0.616	1.04351883	1.159042864
0.000	0.000	0.961	1.42010973	0.892052355
0.000	0.000	0.963	0.900608368	0.827868484
0.000	0.000	0.618	1.462060986	0.908964152
0.000	0.000	0.563	1.323069458	1.071200468
0.000	0.000	0.617	1.454782966	1.100796114
0.000	0.000	0.944	1.325346173	0.734628705
0.000	0.000	0.979	1.848803792	0.902802141
0.000	0.000	0.754	1.062255067	0.982728377
0.000	0.000	0.808	1.004665398	0.925606081
0.000	0.000	0.891	1.446721159	0.9142266
0.000	0.000	0.896	1.854514239	0.974542347
0.000	0.000	0.767	0.998096518	0.735078487
0.000	0.000	0.597	1.272657761	0.725183286
0.000	0.000	0.613	0.957810462	0.778347501
0.000	0.000	0.597	1.027166824	0.823865425
0.000	0.000	1.084	1.007556335	1.097062924
0.000	0.000	1.059	1.797823056	0.775693789
0.000	0.000	0.968	1.729276301	0.62668106
0.000	0.000	0.859	1.309809742	1.151171682
0.000	0.000	0.874	1.273692259	0.715108173
0.000	0.000	0.674	0.974632303	0.71888634
0.000	0.000	0.799	1.702604237	1.154590024
0.000	0.000	0.951	0.994512661	0.660324742
0.000	0.000	0.933	1.104529303	0.958530113
0.000	0.000	0.706	1.467413305	0.785633968
0.000	0.000	1.030	2.160257275	1.082984752
0.000	0.000	1.750	1.28507174	1.198713624
0.000	0.000	0.979	1.711060136	1.128187829
0.000	0.000	0.970	1.764854046	1.332973508
0.000	0.000	0.989	1.457922907	1.231367787

0.000	0.000	0.504
0.000	0.000	0.581
0.000	0.000	0.474
0.000	0.000	0.528
0.000	0.000	0.861
0.000	0.000	0.754
0.000	0.000	0.706
0.000	0.000	0.790
0.000	0.000	0.794
0.000	0.000	0.945
0.000	0.000	0.640
0.000	0.000	0.772
0.000	0.000	1.026
0.000	0.000	0.984
0.000	0.000	0.502
0.000	0.000	0.892
0.000	0.000	0.793
0.000	0.000	0.658
0.000	0.000	0.489
0.000	0.000	0.728
0.000	0.000	0.751
0.000	0.000	0.601
0.000	0.000	0.695
0.000	0.000	0.866
0.000	0.000	0.624
0.000	0.000	0.335
0.000	0.000	0.531
0.000	0.000	0.627
0.000	0.000	0.716
0.000	0.000	0.935
0.000	0.000	0.542
0.000	0.000	0.705
0.000	0.000	0.827
0.000	0.000	0.721
0.000	0.000	0.594
0.000	0.000	0.516
	median	1.000

0.000	0.000	0.949	1.593262268	1.084334098
0.000	0.000	0.930	1.172581298	1.258264742
0.000	0.000	1.634	1.174155535	0.949309585
0.000	0.000	0.829	1.535420321	1.301398822
0.000	0.000	1.035	1.790851437	1.230378267
0.000	0.000	0.994	1.726757523	0.785903837
0.000	0.000	1.061	1.116988261	0.616156164
0.000	0.000	1.457	1.641209014	0.960554131
0.000	0.000	0.756	2.242522377	0.962982953
0.000	0.000	0.913	1.64030945	0.618360095
0.000	0.000	1.073	1.773894661	0.56272208
0.000	0.000	1.085	1.786578509	0.617415553
0.000	0.000	0.897	2.852921333	0.943777268
0.000	0.000	0.997	1.498583187	0.979489947
0.000	0.000	1.069	1.451356092	0.754419107
0.000	0.000	0.447	1.296406243	0.807718257
0.000	0.000	0.782	0.873791211	0.891332704
0.000	0.000	0.702	0.779516934	0.895695588
0.000	0.000	0.736	1.314622408	0.766833086
0.000	0.000	0.768	0.92439167	0.597265326
0.000	0.000	1.006	1.761570638	0.612557909
0.000	0.000	0.646	1.289614537	0.597265326
0.000	0.000	0.745	1.104214456	1.08428912
0.000	0.000	0.689	0.836009535	1.059011379
0.000	0.000	0.657	1.122520578	0.96802051
0.000	0.000	0.827	1.33292853	0.859038366
0.000	0.000	0.776	0.207754239	0.874375928
0.000	0.000	0.561	0.692079342	0.674447893
0.000	0.000	0.741	0.55750461	0.798632663
0.000	0.000	0.694	0.625241758	0.95065893
0.000	0.000	0.791	0.619034768	0.932892547
0.000	0.000	0.507	0.574956146	0.706157514
0.000	0.000	0.663	0.56915396	1.02950569
0.000	0.000	0.499	0.619124725	1.749561463
0.000	0.000	0.565	0.80070166	0.978500427
0.000	0.000	0.622	0.509782755	0.96999955
0.000	0.000	0.642	0.845230063	0.989295192
0.000	0.000	0.764	0.745783295	0.948724868
0.000	0.000	0.930	0.609409436	0.930373769
0.000	0.000	1.030	0.437008051	1.633967526
	median	1.000	0.570863131	0.829262808
			0.572212477	1.035397832
			0.978095624	0.994332749
			0.627535645	1.061035398
			0.593936941	1.457203256
			0.653622993	0.755768452
			1.003418342	0.912922233
			1.484370081	1.073359421
			1.081140647	1.084828858
			0.907254981	0.89664013

1.505015068	0.996581658
1.27738047	1.068996537
1.698961004	0.447353034
1.095083884	0.782305582
1.577070121	0.70179463
1.713578914	0.736068007
1.09841227	0.768497279
1.489182746	1.006027077
0.883821347	0.646381505
0.778527414	0.745063644
1.345972203	0.689290694
0.87729951	0.657221248
2.123734989	0.827283767
2.394998426	0.775738767
1.795664103	0.5612378
1.512751316	0.740655782
1.944451941	0.693518643
1.596545675	0.791166284
2.30589664	0.506994108
1.954212207	0.663428237
1.539738227	0.499347816
1.291323708	0.565060945
1.434669185	0.622408132
1.39562812	0.641658795
1.073719246	0.7635047
1.092520128	0.930238834
1.612917735	1.029820537
1.651239149	
1.499932533	
1.225925426	
1.334367832	
1.925246256	
2.185310125	
1.868753654	
1.905590788	
2.523231233	
1.899968515	
1.735843116	
1.546080151	
1.150586965	
1.430036432	
1.96901003	
0.70228939	
1.342643818	
1.46057662	
1.735888094	
1.812665857	
1.61552647	
1.542661809	
1.797868034	

1.477713309
1.700175415
1.680609904
1.718931318
1.802860613
2.218593982
1.204695723
1.546215086
1.639769712
2.561777538
1.960284262
1.920118742
1.416048217
1.716052714
1.585705933
1.250933297
1.348490982
1.950568974
2.107767733
1.614851797

Counts of nuclei with I_n above the median of the given experiment and calculations corresponding to Figure 4b

exp1	I_n > median	total
eto	122	163
no-tr	18	117

exp2	I_n > median	total
eto	49	63
no-tr	11	58

exp3	I_n > median	total
eto	106	138
no-tr	36	146

Confidence interval calculator	Etoposide	No treatment	Confidence interval calculator (percentage)	Etoposide	No treatment
MLE	0.761	0.2025	MLE	76.1	20.25
Low (adj wald)	0.7145	0.1592	Low (adj wald)	71.45	15.92
High (adj wald)	0.802	0.2467	High (adj wald)	80.2	24.67
Margin	0.0438	0.0437	Margin	4.38	4.37
			Uncertainty+	4.1	4.42
			Uncertainty-	4.65	4.33

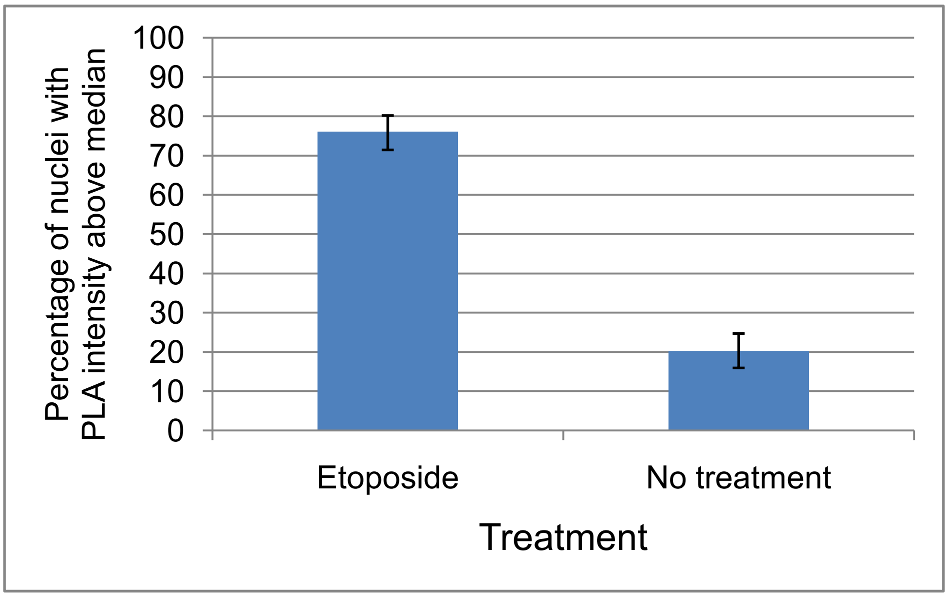

Fisher's Exact Test for Count Data

	etoposide	no-treatment
I_n > median	277	64
I_n < median	87	256

p-value = 0.0000

alternative hypothesis: true odds ratio is ≠

1.0 (two-sided)

95% confidence interval of odds ratio:

8.706994648 ---- 18.664064807

odds ratio = 12.6737650

Counts of nuclei falling into discrete I_n intervals as plotted in Figure 4c

I_n interval	*No treatment*	*Etoposide*	*Total*
0.2	0	0	0
0.4	0	4	4
0.6	27	10	37
0.8	109	16	125
1	100	30	130
1.2	54	55	109
1.4	16	58	74
1.6	6	63	69
1.8	4	49	53
2	1	31	32
2.2	0	18	18
2.4	0	7	7
2.6	0	7	7
2.8	1	5	6
3	0	3	3
3.2	0	2	2
3.4	1	2	3
3.6	0	0	0
3.8	1	0	1
4	1	0	1
More	0	4	4

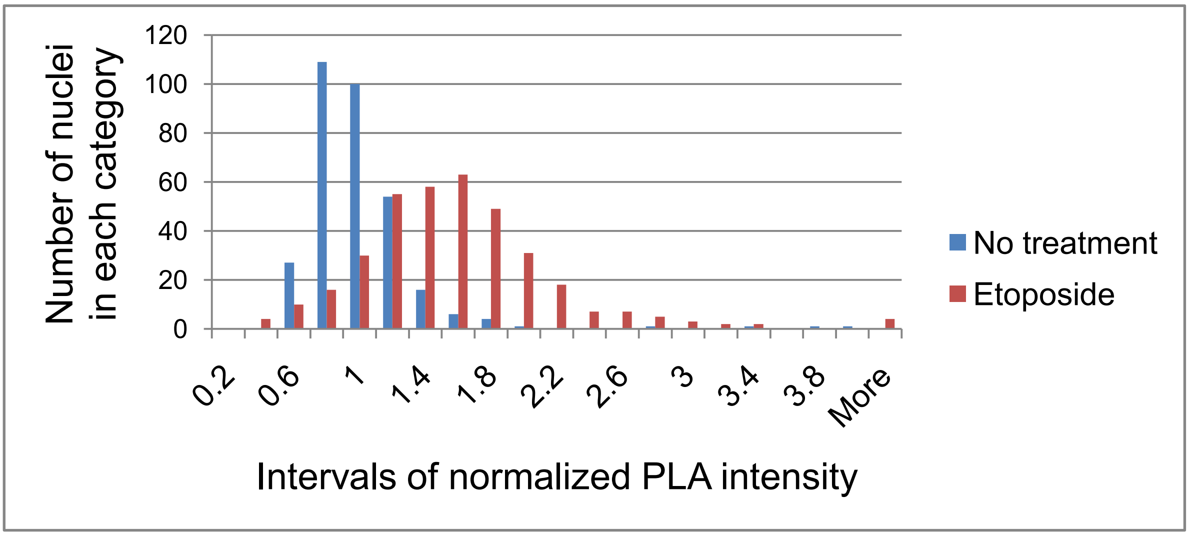

Mean PLA intensities of individual nuclei as identified with macro described in the supplementary methods in one biological replicate for each cell line.

A375+etoposide	A375-no-treatment	A375_merged	HeLa+etoposide	Hela-no-treatment	HeLa_merged
29.813	36.929	29.813	71.733	64.901	71.733
69.97	25.164	69.97	99.983	50.814	99.983
26.894	29.697	26.894	55.357	84.08	55.357
54.412	51.671	54.412	74.129	66.788	74.129
76.431	15.577	76.431	57.883	72.196	57.883
71.794	54.474	71.794	120.149	96.311	120.149
31.421	33.42	31.421	95.45	74.92	95.45
41.048	58.305	41.048	72.787	90.114	72.787
29.73	40.596	29.73	91.763	112.808	91.763
30.342	27.419	30.342	84.471	123.247	84.471
28.086	28.831	28.086	93.431	131.073	93.431
51.321	30.633	51.321	91.167	133.858	91.167
54.078	25.153	54.078	91.41	59.143	91.41
30.353	24.169	30.353	94.611	120.434	94.611
57.842	14.236	57.842	122.033	81.441	122.033
47.673	75.726	47.673	85.616	67.903	85.616
43.147	30.524	43.147	65.071	135.193	65.071
56.15	55.812	56.15	54.71	107.979	54.71
57.79	21.218	57.79	82.358	101.602	82.358
44.464	26.959	44.464	66.81	103.852	66.81
53.377	59.91	53.377	75.017	65.493	75.017
67.742	41.138	67.742	88.898	107.611	88.898
22.026	36.555	22.026	89.425	50.732	89.425
49.429	18.54	49.429	112.181	95.063	112.181
39.452	18.012	39.452	49.32	101.629	49.32
59.139	42.157	59.139	85.262	116.361	85.262
39.224	17.413	39.224	93.389	148.778	93.389
61.399	13.247	61.399	112.195	68.036	112.195
32.439	6.679	32.439	128.785	82.206	128.785
67.954	7.43	67.954	82.512	71.376	82.512
49.725	4.713	49.725	96.23	104.126	96.23
36.166	8.859	36.166	113.281	120.417	113.281
36.862	7.486	36.862	60.172	171.258	60.172
33.744	6.235	33.744	137.56	63.831	137.56
41.939	19.515	41.939	47.901	55.908	47.901
42.881	9.76	42.881	79.068	120.615	79.068
45.014	4.765	45.014	69.719	130.724	69.719
12.121	12.93	12.121	85.302	95.027	85.302
36.63	4.066	36.63	90.234	104.83	90.234
39.867	6.194	39.867	83.99	98.369	83.99
60.41	7.923	60.41	84.717	190.359	84.717
50.225	17.721	50.225	74.634	77.502	74.634
48.561	3.835	48.561	64.755	70.678	64.755
25.695	6.174	25.695	83.165	54.031	83.165
29.002	7.471	29.002	54.21	115.386	54.21
13.828	4.878	13.828	62.542	77.024	62.542

77.234	21.641	77.234	43.37	69.367	43.37
55.501	7.773	55.501	63.711	106.755	63.711
22.703	4.166	22.703	58.969	85.213	58.969
61.059	5.548	61.059	73.822	83.545	73.822
27.168	6.169	27.168	35.079	80.619	35.079
63.146	4.069	63.146	72.192	103.541	72.192
15.41	3.922	15.41	57.563	55.204	57.563
20.366	16.971	20.366	46.716	111.192	46.716
21.752	7.216	21.752	91	84.688	91
33.169	12.13	33.169	64.09	148.387	64.09
19.29	11.362	19.29	58.755	53.033	58.755
23.391	13.167	23.391	69.969	142.855	69.969
42.155	14.032	42.155	56.58	100.005	56.58
38.955	6.541	38.955	80.212	126.4	80.212
31.318	15.761	31.318	69.003	118.453	69.003
26.281	11.374	26.281	60.113	83.462	60.113
27.071	8.168	27.071	63.127	76.216	63.127
20.098	6.426	20.098	52.031	120.814	52.031
12.919	6.247	12.919	51.722	66.123	51.722
10.383	8.877	10.383	83.152	85.279	83.152
14.234	6.312	14.234	57.82	144.991	57.82
10.024	3.364	10.024	91.711	72.241	91.711
6.603	4.715	6.603	52.168	81.736	52.168
6.961	8.283	6.961	53.059	112.991	53.059
8.432	6.855	8.432	40.577	52.01	40.577
18.643	8.661	18.643	69.367	92.793	69.367
11.696	6.156	11.696	106.006	64.425	106.006
6.953	5.675	6.953	95.384	52.633	95.384
13.524	24.853	13.524	67.653	106.281	67.653
19.303	13.218	19.303	65.429	81.146	65.429
10.115	9.258	10.115	153.329	49.236	153.329
8.443	6.34	8.443	79.763	38.182	79.763
10.921	13.965	10.921	178.057	36.433	178.057
9.635		9.635	87.608	53.403	87.608
		36.929	78.107	102.729	78.107
		25.164	76.737	42.644	76.737
		29.697	71.842	30.758	71.842
		51.671	61.807	20.144	61.807
		15.577		49.537	64.901
		54.474		35.676	50.814
		33.42		49.72	84.08
		58.305		48.85	66.788
		40.596		45.623	72.196
		27.419		36.513	96.311
		28.831		60.175	74.92
		30.633		64.463	90.114
		25.153		39.142	112.808
		24.169		41.096	123.247
		14.236		41.822	131.073
		75.726		47.536	133.858

30.524	85.778	59.143
55.812	52.665	120.434
21.218	64.301	81.441
26.959	67.654	67.903
59.91	86.863	135.193
41.138	112.799	107.979
36.555	67.218	101.602
18.54	67.193	103.852
18.012	69.485	65.493
42.157	81.709	107.611
17.413	45.599	50.732
13.247	110.535	95.063
6.679	40.097	101.629
7.43	33.181	116.361
4.713	52.899	148.778
8.859	41.819	68.036
7.486	53.832	82.206
6.235	47.297	71.376
19.515	50.177	104.126
9.76	36.191	120.417
4.765	30.495	171.258
12.93	28.496	63.831
4.066	29.017	55.908
6.194	40.102	120.615
7.923	47.849	130.724
17.721	20.408	95.027
3.835	28.701	104.83
6.174	35.182	98.369
7.471	25.668	190.359
4.878	36.385	77.502
21.641	47.244	70.678
7.773	29.539	54.031
4.166	24.483	115.386
5.548	36.222	77.024
6.169	38.656	69.367
4.069	48.524	106.755
3.922	34.322	85.213
16.971	34.787	83.545
7.216	54.404	80.619
12.13	25.617	103.541
11.362	49.441	55.204
13.167	42.039	111.192
14.032	22.585	84.688
6.541	20.314	148.387
15.761	28.317	53.033
11.374	34.517	142.855
8.168	84.406	100.005
6.426	49.043	126.4
6.247	48.642	118.453
8.877	44.538	83.462

	6.312	42.592	76.216
	3.364	64.697	120.814
	4.715	41.945	66.123
	8.283	58.642	85.279
	6.855	71.886	144.991
	8.661	45.694	72.241
	6.156	57.973	81.736
	5.675	52.872	112.991
	24.853	60.479	52.01
	13.218	45.196	92.793
	9.258	78.597	64.425
	6.34	42.991	52.633
	13.965	54.66	106.281
median	21.752	49.952	81.146
		63.838	49.236
		95.525	38.182
		64.403	36.433
		40.875	53.403
		89.807	102.729
		94.241	42.644
		123.655	30.758
		122.268	20.144
		143.051	49.537
		81.807	35.676
		70.837	49.72
		87.908	48.85
		80.006	45.623
		90.8	36.513
		83.281	60.175
		64.214	64.463
			39.142
			41.096
			41.822
			47.536
			85.778
			52.665
			64.301
			67.654
			86.863
			112.799
			67.218
			67.193
			69.485
			81.709
			45.599
			110.535
			40.097
			33.181
			52.899
			41.819

53.832
47.297
50.177
36.191
30.495
28.496
29.017
40.102
47.849
20.408
28.701
35.182
25.668
36.385
47.244
29.539
24.483
36.222
38.656
48.524
34.322
34.787
54.404
25.617
49.441
42.039
22.585
20.314
28.317
34.517
84.406
49.043
48.642
44.538
42.592
64.697
41.945
58.642
71.886
45.694
57.973
52.872
60.479
45.196
78.597
42.991
54.66
49.952
63.838
95.525

	64.403
	40.875
	89.807
	94.241
	123.655
	122.268
	143.051
	81.807
	70.837
	87.908
	80.006
	90.8
	83.281
	64.214
median	69.602

Mean PLA intensities of individual nuclei normalized to median

A375+etoposide	A375-no-treatment	A375_merged	HeLa+etoposide	Hela-no-treatment	HeLa_merged
1.370586613	1.697728944	1.3705866	1.030616936	0.932458837	1.030616936
3.216715704	1.156859139	3.2167157	1.436496078	0.730065228	1.436496078
1.236392056	1.36525377	1.2363921	0.795336341	1.208011264	0.795336341
2.501471129	2.375459728	2.5014711	1.065041234	0.959570127	1.065041234
3.513745862	0.716118058	3.5137459	0.831628401	1.037269044	0.831628401
3.300570063	2.504321442	3.3005701	1.726229131	1.383738973	1.726229131
1.44451085	1.536410445	1.4445108	1.371368639	1.07640585	1.371368639
1.887090842	2.680443178	1.8870908	1.045760179	1.294704175	1.045760179
1.366770872	1.866311144	1.3667709	1.318396023	1.620758024	1.318396023
1.394906216	1.260527768	1.3949062	1.213628919	1.770739347	1.213628919
1.291191615	1.325441339	1.2911916	1.342360852	1.883178644	1.342360852
2.359369253	1.408284296	2.3593693	1.309833051	1.923191862	1.309833051
2.486116219	1.156353439	2.4861162	1.31332433	0.84973133	1.31332433
1.395411916	1.111116219	1.3954119	1.359314388	1.730323841	1.359314388
2.659157779	0.654468555	2.6591578	1.753297319	1.170095687	1.753297319
2.191660537	3.48133505	2.1916605	1.230079595	0.975589782	1.230079595
1.983587716	1.403273262	1.9835877	0.934901296	1.942372346	0.934901296
2.581371828	2.565833027	2.5813718	0.786040631	1.551377834	0.786040631
2.656767194	0.975450533	2.6567672	1.183270596	1.459756904	1.183270596
2.044133873	1.239380287	2.0441339	0.95988621	1.492083561	0.95988621
2.453889298	2.754229496	2.4538893	1.077799489	0.94096434	1.077799489
3.114288341	1.891228393	3.1142883	1.277233413	1.54609063	1.277233413
1.012596543	1.680535123	1.0125965	1.284805034	0.728887101	1.284805034
2.272388746	0.852335417	2.2723887	1.611749662	1.365808454	1.611749662
1.813718279	0.828061787	1.8137183	0.708600328	1.460144823	0.708600328
2.71878448	1.93807466	2.7187845	1.224993535	1.671805408	1.224993535
1.803236484	0.80052409	1.8032365	1.341757421	2.137553519	1.341757421
2.822682972	0.609001471	2.822683	1.611950806	0.977500647	1.611950806
1.491311144	0.307052225	1.4913111	1.850306026	1.18108675	1.850306026
3.124034572	0.341577786	3.1240346	1.185483176	1.025487773	1.185483176
2.28599669	0.216669732	2.2859967	1.382575213	1.496020229	1.382575213
1.66265171	0.407272894	1.6626517	1.627553806	1.730079595	1.627553806
1.694648768	0.344152262	1.6946488	0.864515387	2.460532743	0.864515387
1.551305627	0.286640309	1.5513056	1.976379989	0.917085716	1.976379989
1.928052593	0.897158882	1.9280526	0.688212982	0.80325278	0.688212982
1.971358955	0.448694373	1.971359	1.136001839	1.732924341	1.136001839
2.069418904	0.219060316	2.0694189	1.001680986	1.878164421	1.001680986
0.557236116	0.594428099	0.5572361	1.225568231	1.365291227	1.225568231
1.683983082	0.18692534	1.6839831	1.296428264	1.506134881	1.296428264
1.832796984	0.284755425	1.832797	1.206718198	1.413307089	1.206718198
2.777215888	0.364242369	2.7772159	1.2171633	2.734964513	1.2171633
2.308983082	0.814683707	2.3089831	1.072296773	1.113502486	1.072296773
2.232484369	0.176305627	2.2324844	0.930361197	1.015459326	0.930361197
1.181270688	0.283835969	1.1812707	1.19486509	0.776285164	1.19486509
1.333302685	0.34346267	1.3333027	0.778856929	1.65779719	0.778856929
0.635711659	0.224255241	0.6357117	0.898566133	1.106634867	0.898566133
3.550662008	0.994897021	3.550662	0.623114278	0.99662366	0.623114278

2.551535491	0.357346451	2.5515355	0.915361628	1.533792132	0.915361628
1.043720118	0.191522619	1.0437201	0.847231401	1.224289532	0.847231401
2.807052225	0.255057006	2.8070522	1.060630442	1.200324703	1.060630442
1.248988599	0.283606105	1.2489886	0.503994138	1.158285681	0.503994138
2.902997426	0.187063259	2.9029974	1.037211574	1.487615298	1.037211574
0.708440603	0.180305259	0.7084406	0.827030832	0.793138128	0.827030832
0.936281721	0.780204119	0.9362817	0.67118761	1.597540301	0.67118761
1	0.33173961	1	1.307433694	1.216746645	1.307433694
1.524871276	0.557649871	1.5248713	0.920806873	2.131935864	0.920806873
0.886815006	0.522342773	0.886815	0.844156777	0.761946496	0.844156777
1.075349393	0.605323648	1.0753494	1.005272837	2.052455389	1.005272837
1.937982714	0.645090107	1.9379827	0.812907675	1.436812161	0.812907675
1.790869805	0.300707981	1.7908698	1.152438148	1.816039769	1.152438148
1.439775653	0.72457705	1.4397757	0.991393925	1.701862015	0.991393925
1.208210739	0.522894446	1.2082107	0.863667711	1.199132209	0.863667711
1.244529239	0.375505701	1.2445292	0.906971064	1.095026005	0.906971064
0.923961015	0.295421111	0.923961	0.747550358	1.735783454	0.747550358
0.593922398	0.287191982	0.5939224	0.74311083	0.950015804	0.74311083
0.477335417	0.408100405	0.4773354	1.194678314	1.225237781	1.194678314
0.654376609	0.290180213	0.6543766	0.830723255	2.083144163	0.830723255
0.460831188	0.154652446	0.4608312	1.317648918	1.037915577	1.317648918
0.303558293	0.216761677	0.3035583	0.749518692	1.174334071	0.749518692
0.32001655	0.380792571	0.3200166	0.762320048	1.623387259	0.762320048
0.387642516	0.315143435	0.3876425	0.582986121	0.747248642	0.582986121
0.857070614	0.398170283	0.8570706	0.99662366	1.333194448	0.99662366
0.537697683	0.283008459	0.5376977	1.523030947	0.925619953	1.523030947
0.319648768	0.26089555	0.3196488	1.37042039	0.756199534	1.37042039
0.621735932	1.142561604	0.6217359	0.971997931	1.526981983	0.971997931
0.887412652	0.60766826	0.8874127	0.940044826	1.165857303	0.940044826
0.465014711	0.425616035	0.4650147	2.202939571	0.707393466	2.202939571
0.388148216	0.291467451	0.3881482	1.145987184	0.54857619	1.145987184
0.502068775	0.64200993	0.5020688	2.558216718	0.523447602	2.558216718
0.442947775		0.4429478	1.258699463	0.767262435	1.258699463
		1.6977289	1.122194765	1.475948967	1.122194765
		1.1568591	1.102511422	0.612683544	1.102511422
		1.3652538	1.032182983	0.441912589	1.032182983
		2.3754597	0.888006092	0.289416971	0.888006092
		0.7161181		0.711718054	0.932458837
		2.5043214		0.512571478	0.730065228
		1.5364104		0.714347289	1.208011264
		2.6804432		0.701847648	0.959570127
		1.8663111		0.655484038	1.037269044
		1.2605278		0.524596994	1.383738973
		1.3254413		0.86455849	1.07640585
		1.4082843		0.926165915	1.294704175
		1.1563534		0.562368897	1.620758024
		1.1111162		0.590442803	1.770739347
		0.6544686		0.600873538	1.883178644
		3.481335		0.68296888	1.923191862
		1.4032733		1.232407115	0.84973133

2.565833	0.756659291	1.730323841
0.9754505	0.923838395	1.170095687
1.2393803	0.972012298	0.975589782
2.7542295	1.247995747	1.942372346
1.8912284	1.620628718	1.551377834
1.6805351	0.965748111	1.459756904
0.8523354	0.965388926	1.492083561
0.8280618	0.998319014	0.94096434
1.9380747	1.173946151	1.54609063
0.8005241	0.65513922	0.728887101
0.6090015	1.588100917	1.365808454
0.3070522	0.576089768	1.460144823
0.3415778	0.476724807	1.671805408
0.2166697	0.760021264	2.137553519
0.4072729	0.600830436	0.977500647
0.3441523	0.773426051	1.18108675
0.2866403	0.679535071	1.025487773
0.8971589	0.720913192	1.496020229
0.4486944	0.51997069	1.730079595
0.2190603	0.438133962	2.460532743
0.5944281	0.409413523	0.917085716
0.1869253	0.41689894	0.80325278
0.2847554	0.576161605	1.732924341
0.3642424	0.687465877	1.878164421
0.8146837	0.293209965	1.365291227
0.1763056	0.41235884	1.506134881
0.283836	0.505473981	1.413307089
0.3434627	0.368782506	2.734964513
0.2242552	0.522757967	1.113502486
0.994897	0.678773598	1.015459326
0.3573465	0.424398724	0.776285164
0.1915226	0.351757133	1.65779719
0.255057	0.52041608	1.106634867
0.2836061	0.555386339	0.99662366
0.1870633	0.697163875	1.533792132
0.1803053	0.493118014	1.224289532
0.7802041	0.499798856	1.200324703
0.3317396	0.781644206	1.158285681
0.5576499	0.368049769	1.487615298
0.5223428	0.710338783	0.793138128
0.6053236	0.603991265	1.597540301
0.6450901	0.324487802	1.216746645
0.300708	0.291859429	2.131935864
0.7245771	0.406841757	0.761946496
0.5228944	0.495919657	2.052455389
0.3755057	1.212695037	1.436812161
0.2954211	0.704620557	1.816039769
0.287192	0.698859228	1.701862015
0.4081004	0.639895405	1.199132209
0.2901802	0.611936439	1.095026005

0.1546524	0.929527887	1.735783454
0.2167617	0.602640729	0.950015804
0.3807926	0.842533261	1.225237781
0.3151434	1.032815149	2.083144163
0.3981703	0.656504123	1.037915577
0.2830085	0.832921468	1.174334071
0.2608955	0.759633344	1.623387259
1.1425616	0.86892618	0.747248642
0.6076683	0.649349157	1.333194448
0.425616	1.129234792	0.925619953
0.2914675	0.617669032	0.756199534
0.6420099	0.785322261	1.526981983
	0.717680526	1.165857303
	0.917186288	0.707393466
	1.372446194	0.54857619
	0.925303871	0.523447602
	0.587267607	0.767262435
	1.290293382	1.475948967
	1.353998448	0.612683544
	1.776601247	0.441912589
	1.756673659	0.289416971
	2.0552714	0.711718054
	1.175354156	0.512571478
	1.017743743	0.714347289
	1.263009684	0.701847648
	1.149478463	0.655484038
	1.304560214	0.524596994
	1.196531709	0.86455849
	0.922588431	0.926165915
		0.562368897
		0.590442803
		0.600873538
		0.68296888
		1.232407115
		0.756659291
		0.923838395
		0.972012298
		1.247995747
		1.620628718
		0.965748111
		0.965388926
		0.998319014
		1.173946151
		0.65513922
		1.588100917
		0.576089768
		0.476724807
		0.760021264
		0.600830436
		0.773426051

0.679535071
0.720913192
0.51997069
0.438133962
0.409413523
0.41689894
0.576161605
0.687465877
0.293209965
0.41235884
0.505473981
0.368782506
0.522757967
0.678773598
0.424398724
0.351757133
0.52041608
0.555386339
0.697163875
0.493118014
0.499798856
0.781644206
0.368049769
0.710338783
0.603991265
0.324487802
0.291859429
0.406841757
0.495919657
1.212695037
0.704620557
0.698859228
0.639895405
0.611936439
0.929527887
0.602640729
0.842533261
1.032815149
0.656504123
0.832921468
0.759633344
0.86892618
0.649349157
1.129234792
0.617669032
0.785322261
0.717680526
0.917186288
1.372446194
0.925303871

0.587267607
1.290293382
1.353998448
1.776601247
1.756673659
2.0552714
1.175354156
1.017743743
1.263009684
1.149478463
1.304560214
1.196531709
0.922588431

Counts of nuclei with I_n above the median in A375

A375	I_n > median	total
eto	57	80
no-tr	22	79

Confidence interval calculator	Etoposide	No treatment	Confidence interval calculator (percentage)	Etoposide	No treatment
MLE	0.7125	0.2785	MLE	71.25	27.85
Low (adj wald)	0.6049	0.1912	ow (adj wald)	60.49	19.12
High (adj wald)	0.8006	0.3863	ligh (adj wald)	80.06	38.63
Margin	0.0978	0.0976	Margin	9.78	9.76
			Uncertainty+	8.81	10.78
			Uncertainty-	10.76	8.73

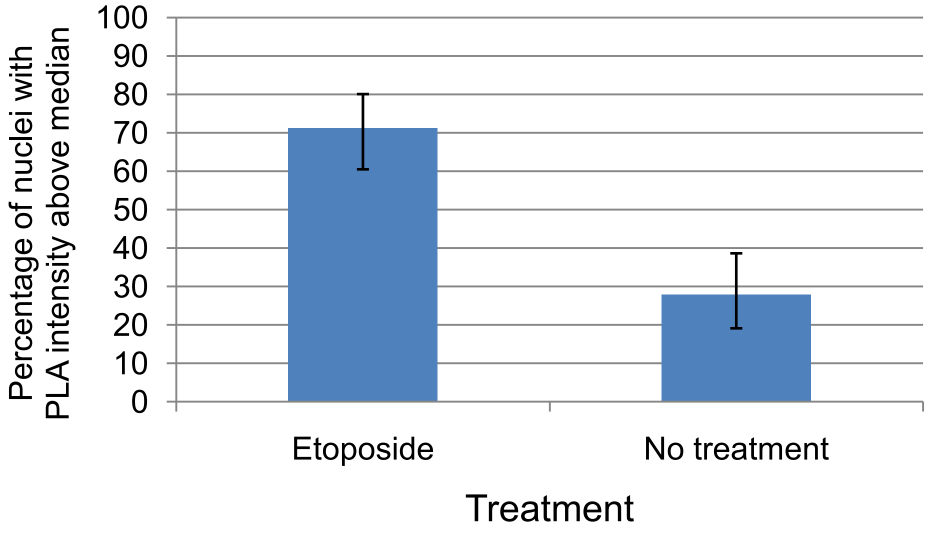

Fisher's Exact Test for Count Data

p-value = 0.0000

alternative hypothesis: true odds ratio is ≠ 1.0 (two-sided)

95% confidence interval of odds ratio:

3.053989075 ---- 13.592542012

odds ratio = 6.3351453

Counts of nuclei with I_n above the median in HeLa

A375	I_n > median	total
eto	51	84
no-tr	79	176

Confidence interval calculator	Etoposide	No treatment	Confidence interval calculator (percentage)	Etoposide	No treatment
MLE	0.6071	0.4489	MLE	60.71	44.89
Low (adj wald)	0.5001	0.3772	Low (adj wald)	50.01	37.72
High (adj wald)	0.7048	0.5227	High (adj wald)	70.48	52.27
Margin	0.1023	0.0727	Margin	10.23	7.27
			Uncertainty+	9.77	7.38
			Uncertainty-	10.7	7.17

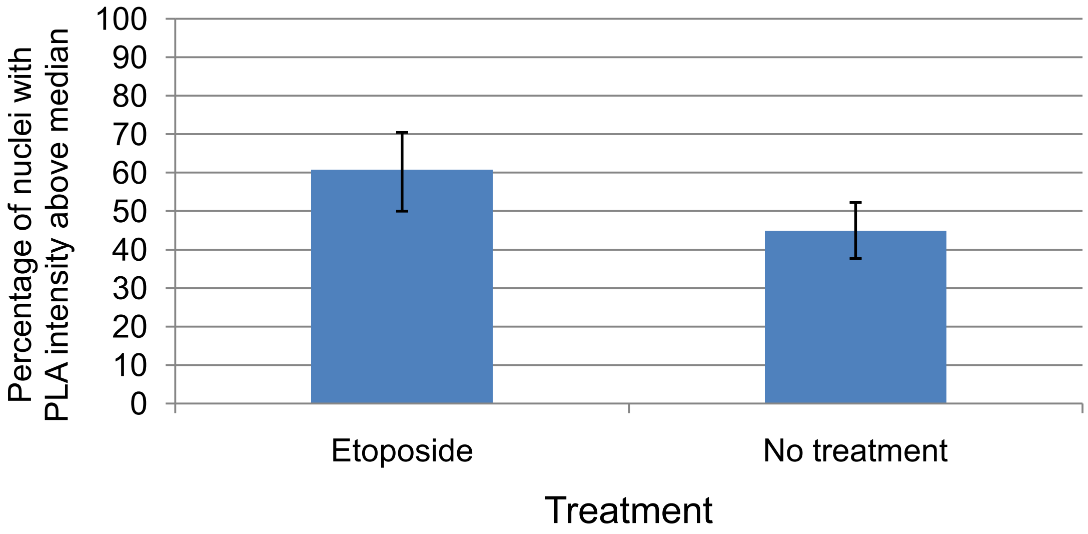

Fisher's Exact Test for Count Data

p-value = 0.0239

alternative hypothesis: true odds ratio is ≠

1.0 (two-sided)

95% confidence interval of odds ratio:

1.082401299 ---- 3.342696439

odds ratio = 1.8928909
